# *Hnrnph1* Is A Quantitative Trait Gene for Methamphetamine Sensitivity

**DOI:** 10.1371/journal.pgen.1005713

**Published:** 2015-12-10

**Authors:** Neema Yazdani, Clarissa C. Parker, Ying Shen, Eric R. Reed, Michael A. Guido, Loren A. Kole, Stacey L. Kirkpatrick, Jackie E. Lim, Greta Sokoloff, Riyan Cheng, W. Evan Johnson, Abraham A. Palmer, Camron D. Bryant

**Affiliations:** 1 Laboratory of Addiction Genetics, Department of Pharmacology and Experimental Therapeutics and Department of Psychiatry, Boston University School of Medicine, Boston, Massachusetts, United States of America; 2 NIGMS Ph.D. Program in Biomolecular Pharmacology, Department of Pharmacology and Experimental Therapeutics, Boston University School of Medicine, Massachusetts, United States of America; 3 Department of Human Genetics, The University of Chicago, Chicago, Illinois, United States of America; 4 Division of Computational Biomedicine, Boston University School of Medicine, Boston, Massachusetts, United States of America; 5 Graduate Program in Bioinformatics, Boston University, Boston, Massachusetts, United States of America; 6 Department of Human Genetics, The University of Chicago, Chicago, Illinois, United States of America; 7 Department of Psychiatry and Behavioral Neuroscience, The University of Chicago, Chicago, Illinois, United States of America; The University of North Carolina at Chapel Hill, UNITED STATES

## Abstract

Psychostimulant addiction is a heritable substance use disorder; however its genetic basis is almost entirely unknown. Quantitative trait locus (QTL) mapping in mice offers a complementary approach to human genome-wide association studies and can facilitate environment control, statistical power, novel gene discovery, and neurobiological mechanisms. We used interval-specific congenic mouse lines carrying various segments of chromosome 11 from the DBA/2J strain on an isogenic C57BL/6J background to positionally clone a 206 kb QTL (50,185,512–50,391,845 bp) that was causally associated with a reduction in the locomotor stimulant response to methamphetamine (2 mg/kg, i.p.; DBA/2J < C57BL/6J)—a non-contingent, drug-induced behavior that is associated with stimulation of the dopaminergic reward circuitry. This chromosomal region contained only two protein coding genes—heterogeneous nuclear ribonucleoprotein, H1 (*Hnrnph1*) and RUN and FYVE domain-containing 1 (*Rufy1*). Transcriptome analysis via mRNA sequencing in the striatum implicated a neurobiological mechanism involving a reduction in mesolimbic innervation and striatal neurotransmission. For instance, *Nr4a2* (nuclear receptor subfamily 4, group A, member 2), a transcription factor crucial for midbrain dopaminergic neuron development, exhibited a 2.1-fold decrease in expression (DBA/2J < C57BL/6J; p 4.2 x 10−^15^). Transcription activator-like effector nucleases (TALENs)-mediated introduction of frameshift deletions in the first coding exon of *Hnrnph1*, but not *Rufy1*, recapitulated the reduced methamphetamine behavioral response, thus identifying *Hnrnph1* as a quantitative trait gene for methamphetamine sensitivity. These results define a novel contribution of *Hnrnph1* to neurobehavioral dysfunction associated with dopaminergic neurotransmission. These findings could have implications for understanding the genetic basis of methamphetamine addiction in humans and the development of novel therapeutics for prevention and treatment of substance abuse and possibly other psychiatric disorders.

## Author Summary

Both genetic and environmental factors can powerfully modulate susceptibility to substance use disorders. Quantitative trait locus (QTL) mapping is an unbiased discovery-based approach that is used to identify novel genetic factors and provide new mechanistic insight into phenotypic variation associated with disease. In this study, we focused on the genetic basis of variation in sensitivity to the acute locomotor stimulant response to methamphetamine which is a behavioral phenotype in rodents that is associated with stimulated dopamine release and activation of the brain reward circuitry involved in addiction. Using brute force monitoring of recombination events associated with changes in behavior, we fortuitously narrowed the genotype-phenotype association down to just two genes that we subsequently targeted using a contemporary genome editing approach. The gene that we validated–*Hnrnph1* –is an RNA binding protein that did not have any previously known function in psychostimulant behavior or psychostimulant addiction. Our behavioral data combined with our gene expression results provide a compelling rationale for a new line of investigation regarding *Hnrnph1* and its role in neural development and plasticity associated with the addictions and perhaps other dopamine-dependent psychiatric disorders.

## Introduction

Substance use disorders (SUDs) involving psychostimulants such as cocaine and methamphetamine (MA) are heritable; however, their major genetic determinants remain poorly defined [1–4]. In particular, genome-wide association studies (GWAS) of psychostimulant abuse have yet to discover the underlying genetic factors or causal sequence variants. SUDs involve multiple discrete steps including initial use, escalation, withdrawal, and relapse, each of which is believed to have a distinct genetic architecture. Therefore, we and others have used model organisms to explore the genetic basis of intermediate phenotypes, including initial drug sensitivity [5]. Model systems have great potential for studying addiction-relevant intermediate phenotypes [6] because they provide exquisite control over environmental conditions, including exposure to psychostimulants.

Psychostimulants activate the mesocorticolimbic reward circuitry in humans [7] and stimulate locomotor activity in mice [8]. The primary molecular targets of psychostimulants are the membrane-spanning monoaminergic transporters. Amphetamines act as substrates and cause reverse transport and synaptic efflux of dopamine, norepinephrine, and serotonin [9–11]. Sensitivity to the locomotor stimulant response to MA is heritable and may share a genetic basis with the addictive, neurotoxic, and therapeutic properties of amphetamines [8, 12–15]. More broadly, determining the genetic basis of sensitivity to amphetamines may provide insight into the neurobiology of other conditions involving perturbations in dopaminergic signaling, including attention deficit hyperactive disorder (ADHD), schizophrenia, and Parkinson’s disease [16]. This hypothesis is supported by our recent identification of a genetic correlation between alleles that increased amphetamine-induced euphoria and alleles that decreased risk of schizophrenia and ADHD [17].

We and others have reported several quantitative trait loci (QTLs) in mice that influence MA sensitivity [12, 18–24]. A distinct advantage of QTL analysis is that chromosomal regions can eventually be mapped to their causal polymorphisms. However, obtaining gene-level and nucleotide-level resolution can be extremely challenging when beginning with a lowly recombinant population such as an F_2_ cross. A classical approach is to fine map QTLs derived from an F_2_ cross using successively smaller congenic strains. Whereas this approach is efficient for Mendelian alleles, there are only a few examples in which this approach has been successful in identifying alleles for more complex, polygenic traits, such as histocompatibility [25], substance abuse [26] and depressive-like behavior [27].

In the present study, we fine mapped a QTL on chromosome 11 that modulates methamphetamine sensitivity and that segregates between C57BL/6J (B6) and DBA/2J (D2) inbred strains [12, 20]. We used interval-specific congenic lines in which successively smaller D2-derived segments were introgressed onto a B6 background [28]. We also conducted transcriptome analysis of brain tissue from a congenic line that captured the QTL for reduced MA sensitivity. Our transcriptome analysis focused on the striatum, which is a brain region important for psychostimulant-induced locomotor activity and reward [29]. We used GeneNetwork [30] and *in silico* expression QTL (eQTL) analysis of several brain regions to identify *cis*- and *trans*-eQTLs that may explain changes in the transcriptome caused by this QTL. Finally, to identify the quantitative trait gene responsible for reduced MA sensitivity, we used transcription activator-like effector nucleases (TALENs) to introduce frameshift deletions in the first coding exon of each positional candidate gene [31].

## Results

### Identification of a 206 kb critical interval for reduced MA sensitivity

**Fig 1:**
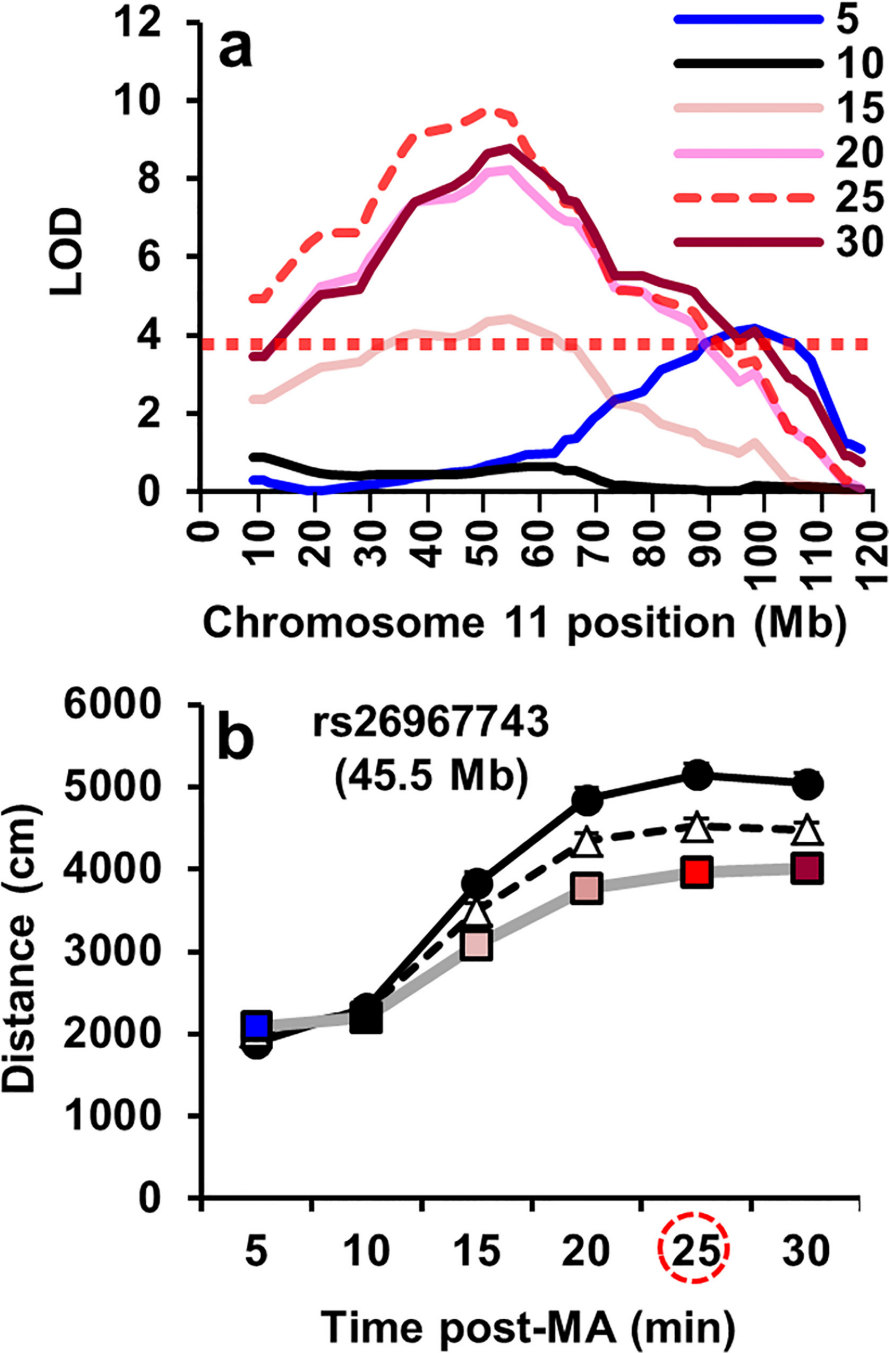
QTL for reduced MA sensitivity (D2 < B6) in B6 x D2-F_2_ mice. **(a):** We previously published a genome-wide a significant QTL on chromosome 11 for MA-induced locomotor activity from the same B6 x D2-F_2_ dataset (N = 676) that was significant when the data were summed from 15–30 min and 0–30 min but not when the data were summed from 0–15 min [20]. To further dissect the time dependency of this locus, we generated LOD scores from the same mice in six, 5-min time bins over 30 min. The x-axis represents the physical distance (Mb) of the marker on chromosome 11 (mm9). The y-axis represents the LOD score. The dashed, horizontal line represents the genome-wide significance level derived from 1,000 permutations. The dark blue QTL trace (5 min) denotes a distal locus (90 Mb) in which inheritance of the D2 allele caused an *increase* in locomotor activity relative to the B6 allele that was most likely not associated with MA treatment (see QTL for Days 1 and 2 in response to saline; S1 Fig; D2 > B6). The remaining red- and pink-shaded QTL traces denote a separate locus (50 Mb) that was specific for MA treatment on Day 3 in which inheritance of the D2 allele caused a *decrease* in MA-induced locomotor activity. The dashed QTL trace indicates the time bin containing the peak LOD score. (**b):** The effect plot for the marker nearest the peak LOD score is shown for the six, 5-min time bins. Data are sorted by genotype for each time bin. The time bin with the most significant LOD score is circled. B6 = homozygous for B6 allele (circles); H = heterozygous (triangles); D2 = homozygous for D2 allele (colored squares). Data are presented as the mean ± S.E.M.

**Fig 2:**
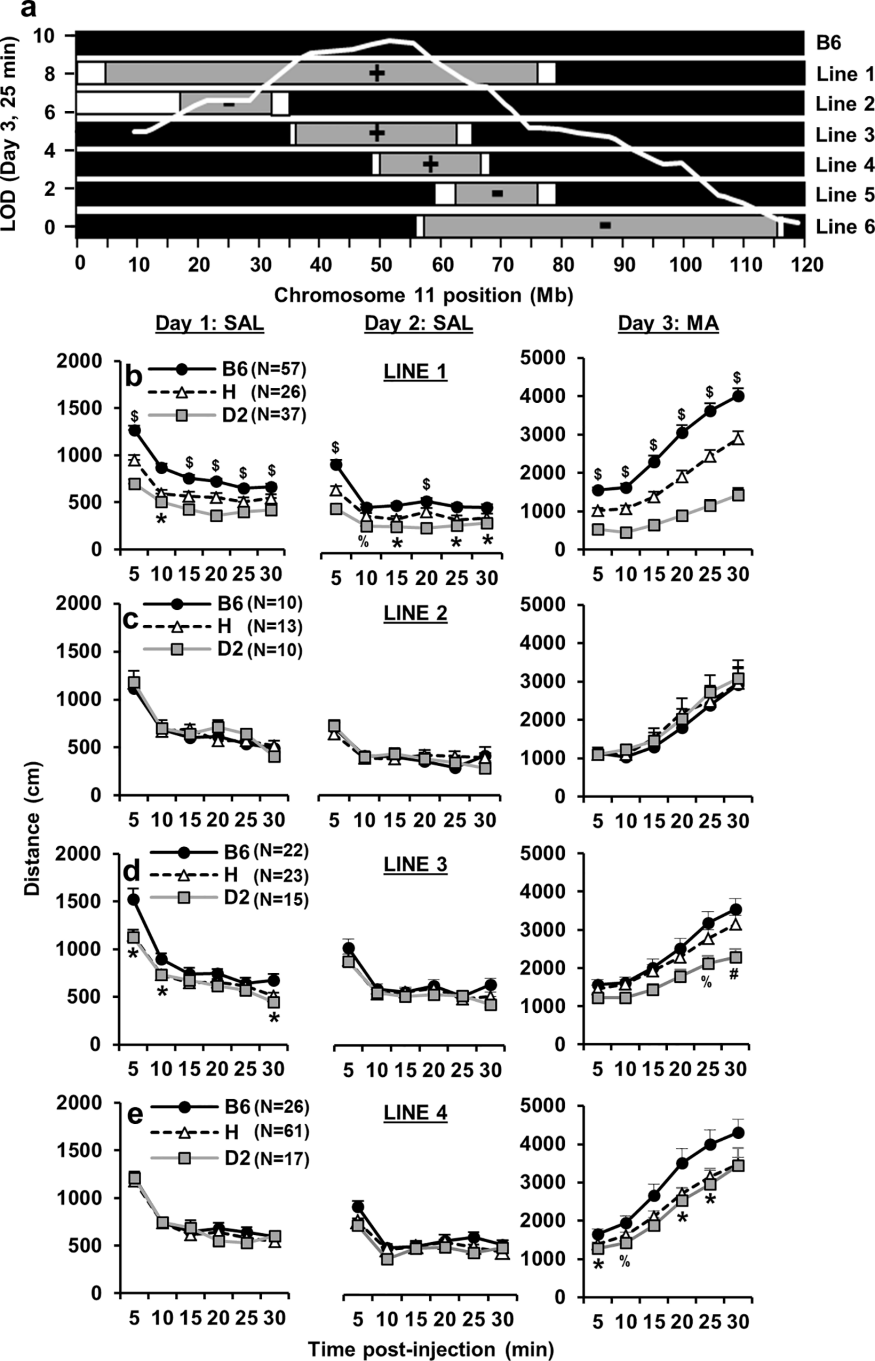
Congenic analysis identifies Line 4 for fine mapping. Statistical results are provided in S3 Table and described in the Supplementary Information. **(a):** Lines 1–6 possessed either one (heterozygous; “H”) or two copies (homozygous, “D2”) of a chromosome 11 interval from the D2 inbred strain (gray region) on an isogenic B6 background (black region; denotes the genotype for the rest of the genome). The white regions represent transitional regions that were not genotyped. The x-axis represents the physical position (Mb) of the SNP marker. The SNP markers that were used to genotype Lines 1–6 are listed in S1 Table. The y-axis represents the LOD score for the F_2_-derived QTL that was causally associated with reduced MA sensitivity on Day 3 (D2 < B6; Fig 1; 25 min bin). (+) = congenic line captured the QTL for reduced MA sensitivity on Day 3. (-) = congenic line failed to capture the QTL. **(b-e):** The three columns represent the phenotypes for Days 1, 2, and 3. The four rows represent Lines 1–4. The negative results for Lines 5 and 6 (-) are shown in S2 Fig and described in S1 Text. “*” indicates a dominant effect of the D2 allele (D2 = H < B6) or H < B6. **“$”** indicates an additive effect (D2 < H < B6). **“#”** indicates a recessive effect (D2 < H = B6). “**%**” indicates that B6 and D2 differ from each other but not from H. “**&**” indicates that H and D2 differ from each other but not from B6. Data are represented as the mean ± S.E.M. p < 0.05 was considered significant. We estimated the narrow-sense heritability of the QTLs (h^2^) for Line 3 and Line 4 (25 min) based on the intraclass correlation coefficient using the phenotypic variances from homozygous D2 versus homozygous B6 mice according to the following formula: h^2^ = (between-genotype variance) / (between-genotype variance + within-genotype variance). For Line 3, h^2^ = 0.35; for Line 4, h^2^ = 0.08. Although these h^2^ estimates do not contain confidence intervals, the differences in h^2^ values combined with the different modes of inheritance suggest that Line 3 and Line 4 possess different QTLs.

Several genome-wide significant QTLs that influenced MA sensitivity were previously reported in this B6 x D2-F_2_ cross, including QTLs on chromosomes 1, 8, 9, 11, 15, and 16 [20]. Here, we further dissected the chromosome 11 QTL (peak = 50 Mb; D2 < B6) into 5 min bins and identified a peak LOD score at 25 min post-MA administration (Fig 1). We then produced interval-specific congenic lines to fine map this QTL. The genomic intervals (Mb) for the congenic lines and the peak F_2_-derived QTL are illustrated in Fig 2A and the SNP markers that defined the congenic intervals for Lines 1–6 are listed in S1 Table. As shown in Fig 2B–2E, some of the congenic lines captured a QTL that reduced MA sensitivity whereas others did not (see also S2A and S2B Fig and S1 Text). Whether or not a strain captured a QTL is indicated by a + or–sign in Fig 2A.

**Fig 3:**
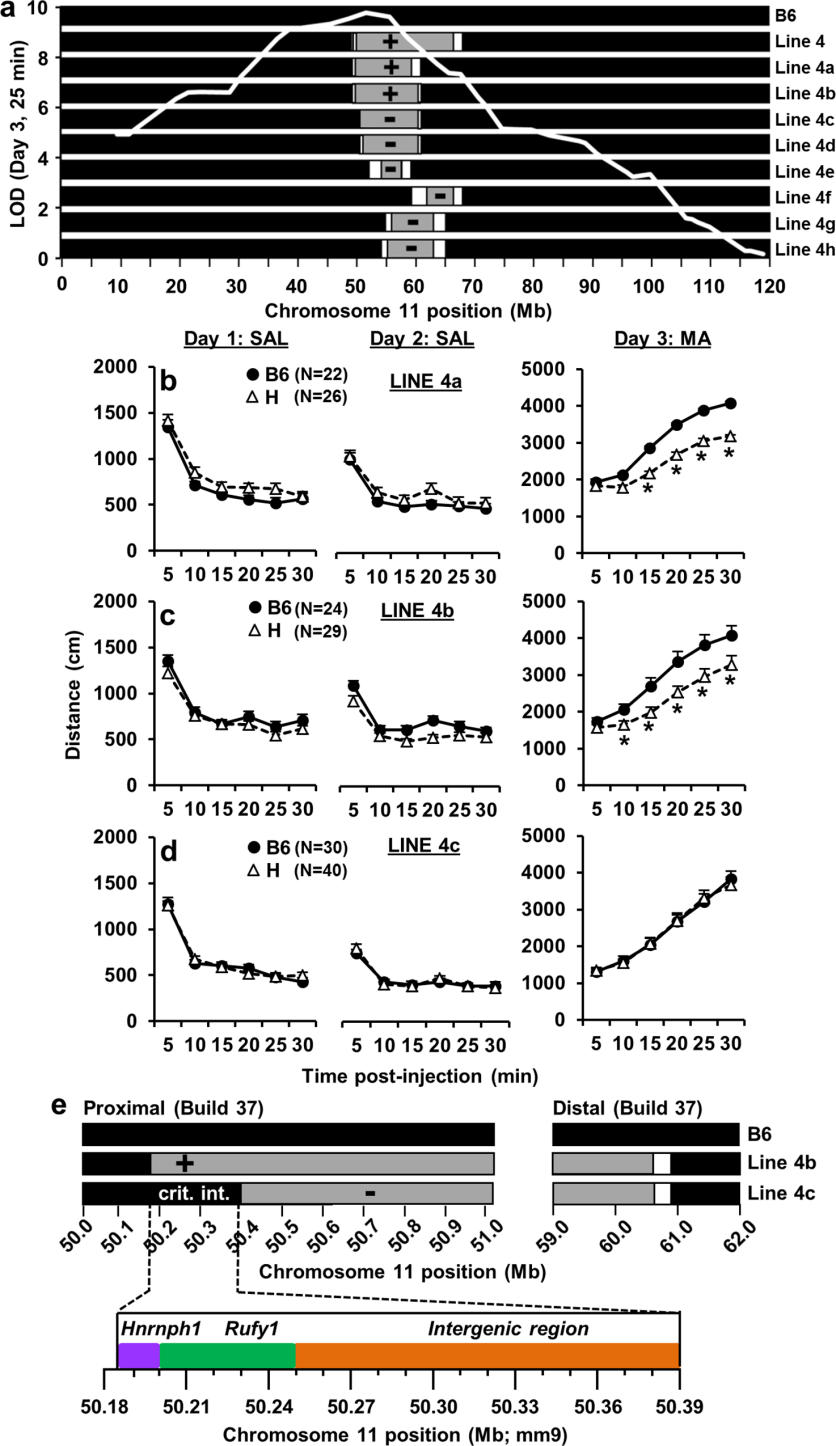
Analysis of subcongenic lines from Line 4 reveals a 206 kb critical interval for reduced MA sensitivity. Statistical results are provided in S3 Table and described in the Supplementary Information. **(a):** Lines 4a-4h possessed heterozygous (H) intervals of B6 and D2 origin (gray regions) on an isogenic B6 background (black; denotes the genotype for the rest of the genome). The white regions represent transitional regions that were not genotyped. The x-axis represents the physical position (Mb) on chromosome 11. The SNP markers used for genotyping Lines 4a-4h are listed in S2 Table. The y-axis represents the peak LOD score for the F_2_-derived QTL causing reduced MA sensitivity on Day 3 (Fig 1C; 25 min; white QTL trace). (+) = subcongenic line captured the QTL for reduced MA sensitivity. (-) = subcongenic line failed to capture the QTL. **(b-d):** The three columns represent the phenotypes for Days 1, 2, and 3. The three rows represent Lines 4a-4c. The negative results for Lines 4d-4h (-) are shown in S3 Fig and described in S1 Text “*” = significantly different from B6 (p < 0.05). Data are represented as the mean ± S.E.M. **(e):** The proximal boundary of Line 4b (+) and the proximal boundary of Line 4c (-) define the 206 Kb critical interval (crit. int.; 50,185,512–50,391,845 bp; mm9; S2 Table) which contains two protein coding genes—*Hnrnph1* and *Rufy1*.

Congenic Line 4 was the smallest congenic that captured a QTL for reduced MA sensitivity. Therefore, we produced subcongenic lines from Line 4, as shown in Fig 3A. The SNP markers that defined the congenic intervals for Lines 4a-4h are listed in S2 Table. Production and analysis of these congenic lines was more efficient because the D2-derived allele was dominant. Therefore all lines shown in Fig 3 were heterozygous for the D2-derived congenic interval. Once again, some but not all of the congenic lines captured the QTL inherited from Line 4 (Figs 3B, 3C, 3D and S3, S3 Table and S1 Text). Based on the observation that Line 4b but not 4c captured the QTL, we were able to define a 206 kb critical interval (Fig 3E). The first proximal SNP in Lines 4b was rs29424921 and first proximal SNP in Line 4c was rs29442500. The physical location of these SNPs defined the boundaries of the critical interval (50,185,512–50,391,845 bp; S2 Table). This interval contains only two protein coding genes: *Hnrnph1* (heterogeneous nuclear ribonucleoprotein) and *Rufy1* (RUN and FYVE domain containing 1; Fig 3E and S4 Table).

Using Line 4c to define the distal boundary presumes that our analysis of Line 4c was powerful enough to detect the QTL if it were present. We used data generated from Line 4b to estimate the QTL effect size; based on this estimate, a sample size of N = 25 per group would be required to achieve 80% power to detect this QTL in Line 4c. We phenotyped an even larger number of mice from Line 4c (N = 30–40 per genotype), but did not detect the QTL (Fig 3D). Therefore, we can confidently interpret the negative results from Line 4c. Further negative results obtained from five additional subcongenic lines also support the critical interval as defined in Fig 3E (see S3 Fig and S3 Table).

### Residual heterozygosity

Studies of congenic lines can be confounded by residual heterozygosity that lies outside of the congenic region. In order to address this concern, we genotyped individuals from Line 4 subcongenics at 882 SNPs using a SNP genotyping microarray. Although we did identify a single D2-derived SNP on chromosome 3, it was observed both in wild-type and heterozygous congenic mice and was not associated with the locomotor response to MA (see S4 Fig and S1 Text). Based on these results we rejected the possibility that the differences in the congenic lines were due to residual heterozygosity.

### Transcriptome of Line 4a

**Fig 4:**
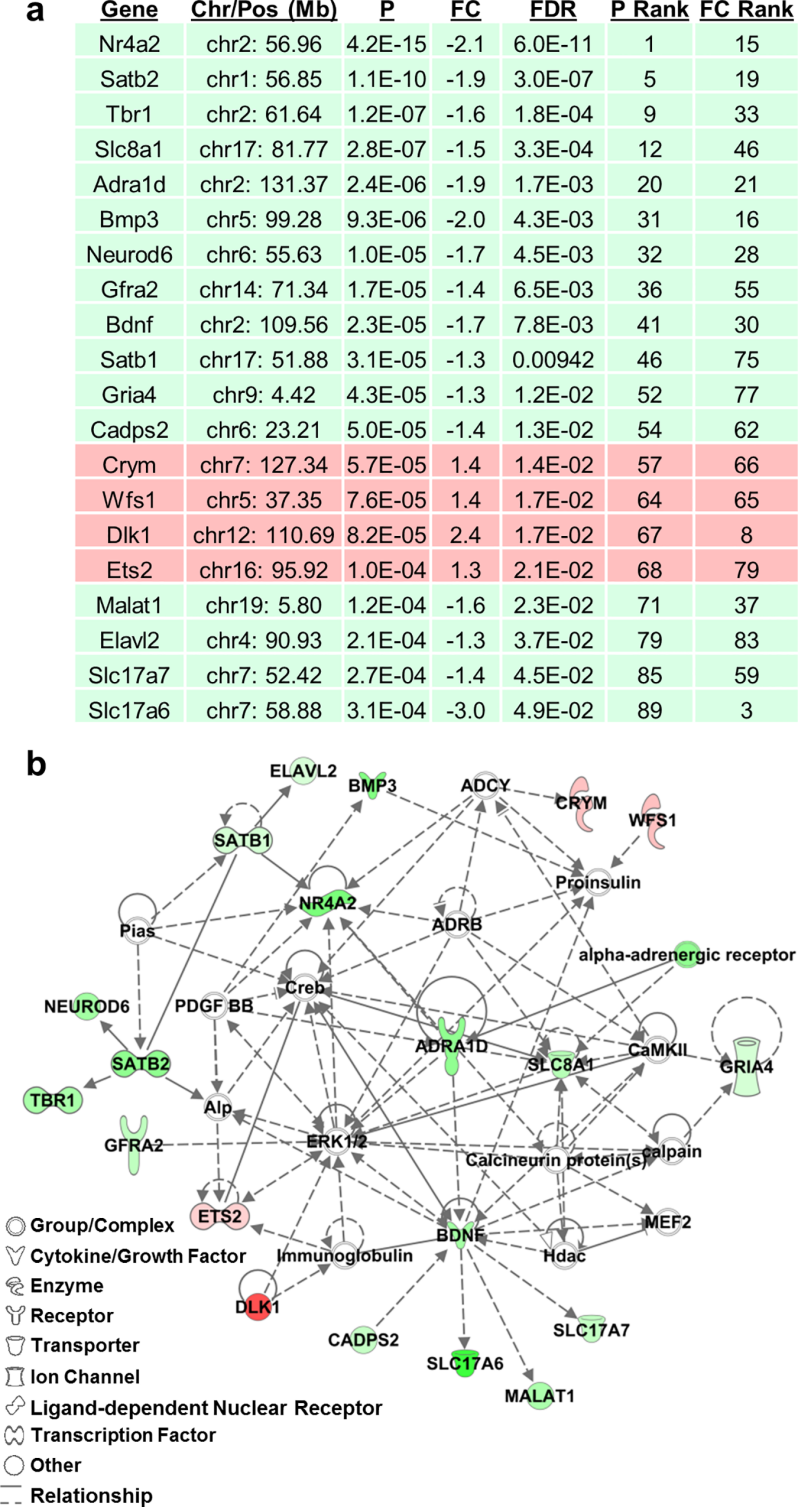
Transcriptome analysis of Line 4a identifies “Cellular Development, Nervous System Development and Function, Behavior” as the top IPA network. **(a, b):** 17 downregulated genes (green) and four upregulated genes (red) were identified in the IPA network (S10 Table). Genes in the network diagram that lack any color were included by the IPA algorithm to facilitate connectivity. Chromosome and position (Chr/Pos; mm9) of each gene is shown. P = p-value of differential expression in Line 4a; FC = fold-change in expression; FDR = false discovery rate (< 0.05; 5%), P Rank = rank in p-value (#1 = lowest p-value out of 91 genes); FC Rank = rank in fold-change (#1 = largest fold-change out of 91 genes).

In an effort to understand the molecular impact of this QTL, we used RNA-seq to identify gene expression differences in the striatum of naïve Line 4a congenics versus their naïve B6 littermates. We identified between 91 differentially expressed genes with an FDR of 5% and 174 differentially expressed genes with and FDR of 20%. The majority of these genes were downregulated in Line 4a (S6 Table). Notably, *Nr4a2* (*Nurr1*) was the most significant, demonstrating a 2.1-fold decrease in expression (p = 4.2 x 10^−15^; Fig 4). Decreased *Nurr1* expression in Line 4a was confirmed using qPCR (S5A Fig and S7 Table).

We used the Ingenuity Pathway Analysis (IPA; Ingenuity Systems, Redwood City, CA, USA; www.qiagen.com/ingenuity) software in conjunction with the genes we identified with an FDR of 5% to explore pathways that were enriched for these genes. The top three canonical pathways that we identified included the neuronal functions Glutamate Receptor Signaling, G_αq_ Signaling, and G-Protein Coupled Receptor Signaling (S8 Table). Neither transcriptome nor qPCR analysis detected any significant difference in gene- or exon-level expression of *Hnrnph1* or *Rufy1* (S5B, S5C and S6 Figs). The most strongly implicated IPA network was, “Cellular Development, Nervous System Development and Function, Behavior”. This network consists of several downregulated genes involved in neural development, maintenance, and signaling (Fig 4), including *Bdnf*, which was downregulated and connected to several downregulated genes involved in synaptic transmission, including *Malat1*, the vesicular glutamate transporters VGLUT1 (*Slc17a7*) and VGLUT2 (*Slc17a6*), as well as the AMPA-4 receptor subunit (*Gria4*), alpha-1d adrenergic receptor (*Adra1d*), and calcium-dependent secretion activator 2 (*Cadps2*). The top “Diseases and Functions” annotations included Huntington’s disease, nervous system coordination, and disorder of basal ganglia (S9 Table), further supporting dysfunction in striatal innervation and signaling. *Htt* (huntingtin) was the top predicted upstream transcriptional regulator followed by *Creb1* (cyclic AMP response element binding protein) which together accounted for 23 (25%) of the 91 differentially expressed genes (S7 Fig).

Gene Ontology (GO) pathways identified via WebGestalt [32, 33] complemented the IPA results and generally indicate neuronal dysfunction. The top biological process was synaptic transmission and signaling processes, the top molecular functions involved membrane proteins including transporters and g protein-coupled receptors and the top cellular components were associated with neuronal synapses (Table 1).

**Table 1:** WebGestalt-Gene Onotology (GO) analysis of differentially expressed genes in the striatum of Line 4a. GO enrichment analysis of our gene list (91 genes, FDR < 5%) was performed using a hypergenometric statistical procedure and multiple testing adjustment (Adj P). A minimum of two genes was required per category.

**Biological Process**	**GO ID**	**P**	**Adj P**	**# of Genes**
Synaptic transmission	0007268	6.40E-12	2.07E-09	16
Multicellular organismal signaling	0035637	3.36E-12	2.07E-09	18
Transmission of nerve impulse	0019226	1.84E-11	3.97E-09	17
Cell-cell signaling	0007267	1.09E-09	1.77E-07	17
Single-organism process	0044699	1.91E-08	2.48E-06	54
Multicellular organismal process	0032501	5.60E-07	4.54E-05	43
Biological regulation	0065007	5.46E-07	4.54E-05	57
Single-multicellular organism process	0044707	5.25E-07	4.54E-05	43
Single organism signaling	0044700	1.25E-06	8.10E-05	39
Signaling	0023052	1.25E-06	8.10E-05	39
**Molecular Function**	**GO ID**	**P**	**Adj P**	**# of Genes**
Transporter activity	0005215	1.74E-06	2.00E-04	16
Transmembrane transporter activity	0022857	1.59E-05	1.10E-03	13
Secondary active transmembrane transporter activity	0015291	4.94E-05	1.80E-03	6
Alpha1-adrenergic receptor activity	0004937	4.10E-05	1.80E-03	2
Substrate-specific transporter activity	0022892	1.00E-04	2.40E-03	12
Substrate-specific transmembrane transporter activity	0022891	1.00E-04	2.40E-03	11
Anion transmembrane transporter activity	0008509	2.00E-04	3.60E-03	6
Transmembrane transporter activity	0015075	4.00E-04	6.30E-03	10
Adrenergic receptor activity	0004935	5.00E-04	6.60E-03	2
**Cellular Component**	**GO ID**	**P**	**Adj P**	**# of Genes**
Cell junction	0030054	8.26E-08	9.17E-06	15
Synapse	0045202	1.07E-06	5.94E-05	12
Plasma membrane	0005886	3.63E-06	1.00E-04	31
Cell periphery	0071944	6.17E-06	2.00E-04	31
Synapse part	0044456	1.87E-05	4.00E-04	9
Cell part	0005623	2.00E-04	3.20E-03	66
Neuron spine	0044309	4.00E-04	4.90E-03	5
Dendritic spine	0043197	4.00E-04	4.90E-03	5
Postsynaptic membrane	0045211	6.00E-04	6.70E-03	5

### eQTLs associated with differentially expressed genes in Line 4a

In order to identify genetic polymorphisms associated with changes in gene expression observed in the congenic region of Line 4a, we used GeneNetwork [30] to identify both *cis*- and *trans*-eQTLs that originated from B6/D2 polymorphisms within the Line 4a congenic region (FDR < 20%; S6 Table). We identified several *trans*-QTLs caused by SNPs within the Line 4a region, including a link between genetic variation in *Hnrnph1* and differential expression of *Ipcef1* (Tables 2 and S6) [30], a gene that lies within *Oprm1* (mu opioid receptor) and is transcribed in the reverse direction. These observations support the gene expression differences we observed using RNA-seq and indicate that our QTL regulates the expression of numerous other genes outside of the QTL interval.

**Table 2:** Differentially expressed genes in Line 4a (FDR < 20%) that possessed *cis*- or *trans*-eQTLs in GeneNetwork (GN). Differentially expressed genes (DEGs) are shown from our striatal RNA-seq dataset (FDR < 20%) that possess known eQTLs from GeneNetwork caused by genetic variation within the Line 4a locus (chromosome 11: 50–60 Mb). **With regard to DEGs from our dataset:** Chr/Pos = chromosome and position of each DEG; FC = fold-change; P = p-value; Q = q-value. **With regard to eQTLs identified in GeneNetwork:** The GeneNetwork genes associated with differential expression of DEGs from our dataset are listed [LRS ≥ 13.8 (LOD ≥ 3)]; NAc = nucleus accumbens; Str = striatum; NCTX = neocortex; PFC = prefrontal cortex; HC = hippocampus; LRS = likelihood ratio statistic; GN = GeneNetwork. eQTLs were identified from the following datasets: UTHSC Hippocampus Illumina v6.1 All Combined (Nov12) RankInv Database; Hippocampus Consortium M430v2 (Jun06) PDNN Database UTHSC Hippocampus Illumina v6.1 NON (Sep09) RankInv Database; Hippocampus Consortium M430v2 (Jun06) RMA Database; BIDMC-UTHSC Dev Neocortex P3 ILMv6.2 (Nov11) RankInv Database; BIDMC-UTHSC Dev Neocortex P14 ILMv6.2 (Nov11) RankInv Database; HQF BXD Neocortex ILM6v1.1 (Dec10v2) RankInv Database; HQF BXD Neocortex ILM6v1.1 (Feb08) RankInv Database; VCU BXD NAc Sal M430 2.0 (Oct07) RMA Database; HQF Striatum Affy Mouse Exon 1.0ST Gene Level (Dec09) RMA Database; HQF BXD Striatum ILM6.1 (Dec10v2) RankInv Database; HBP Rosen Striatum M430V2 (Apr05) RMA Clean Database

Gene ID for DEG (RNA-seq)	Gene name (RNA-seq)	Chr/Pos of DEG (Mb)	Log_2_FC of DEG (± FC)	P-value of DEG	FDR of DEG	Associated GeneNetwork genes within Line 4a region	eQTL LRS	Brain Region
*Slc8a1*	solute carrier family 8 (sodium/calcium exchanger), member 1	17:81.77	-0.59 (-1.50)	2.9x10^-7^	3.3x10^-4^	*Olfr51* (50.8 Mb)	19.1	NTCX
*Satb1*	special AT-rich sequence binding protein 1	17:51.87	-0.35 (-1.27)	3.1x10^-5^	9.4x10^-3^	*B130040O20Rik* (49.8 Mb)	18.2	NCTX
*Obscn*	obscurin	11:50.89	1.23 (+2.34)	1.9x10^-5^	0.01	*2610507I01Rik*,*Mrpl55*,*D130047N11Rik*,*Gja12*,*Guk1*, *2810021J22Rik* (50–59 Mb)	20–82	NAc, Str, NCTX, PFC, Hipp
*Megf11*	multiple EGF-like-domains 11	9: 64.23	-0.46 (-1.38)	4.5x10^-5^	0.01	*Mprip* (59.5 Mb),*Tom1l2* (60.0 Mb)	14.3, 14.4	NAc, NCTX
*Malat1*	metastasis associated lung adenocarcinoma transcript 1	19:5.79	-0.68 (1.60)	1.2x10^-4^	0.023	*Il3* (54.0 Mb)	15.1	NCTX
*Mkx*	mohawk homeobox	18:6.93	-0.47 (-1.38)	5.1x10^-4^	0.07	*Olfr323* (58.4 Mb)	16.0	NCTX
*Hs3st2*	heparan sulfate 3-O-sulfotransferase 2	7:128.53	-0.52 (-1.43)	8.5x10^-4^	0.11	*Cops3* (59.6 Mb)	14.4	PFC
*Ipcef1*	interaction protein for cytohesin exchange factors 1	10:3.37	-0.57 (-1.48)	1.1x10^-3^	0.12	***Hnrnph1*** (50.2 Mb), *G3bp1* (55.3 Mb)	15.1, 18.8	NCTX
*Tgm2*	transglutaminase 2, C polypeptide	2:157.95	0.46 (+1.37)	1.4x10^-3^	0.15	*N4bp3* (51.5 Mb)	15.7	PFC
*9230009I02Rik*		11:50.89	-0.94 (-1.92)	1.6x10^-3^	0.16	*Agxt2l2*,*D11Ertd497e*, *Col23a1*,*Hnrpab*,*Lyrm7*, *G3bp1*,*Clk4*,*Damts2*, *Gria1*,*Zfp354a* (51–57 Mb)	17–66	NCTX
*Ubash3b*	ubiquitin associated and SH3 domain containing, B	9:40.82	-0.61 (-1.54)	1.7x10^-3^	0.17	*Zfp2* (50.7 Mb)	14.9	HC
*Ablim2*	actin-binding LIM protein 2	5:36.10	0.21 (+1.16)	2.4x10^-3^	0.20	*Olfr54* (36.2 Mb)	14	Str

### Recapitulation of the congenic QTL phenotype in mice heterozygous for a frameshift deletion in *Hnrnph1*, but not *Rufy1*

**Fig 5:**
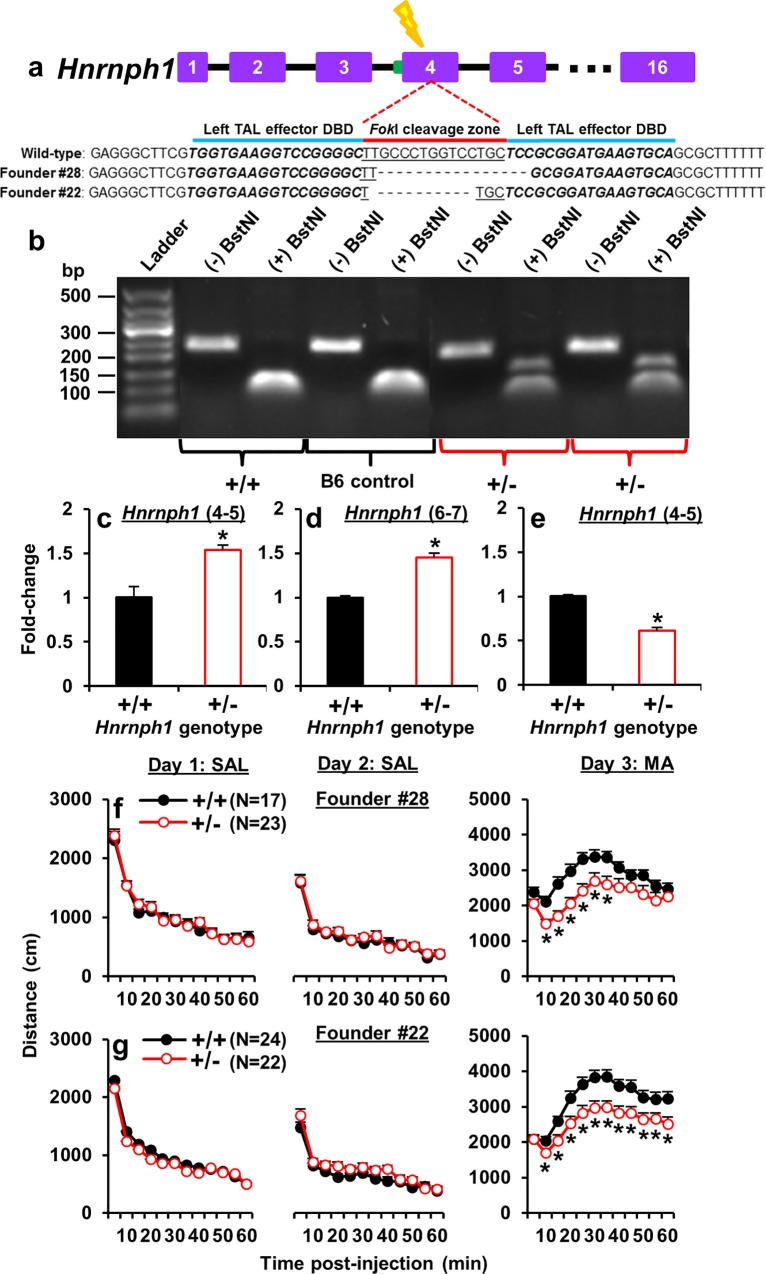
TALENS-targeted frameshift deletions in *Hnrnph*1 ^+/-^ mice reveal *Hnrnph1* as a quantitative trait gene for MA sensitivity. **(a):** Left TAL effector (50,191,867–50,191,883 bp) and right TAL effector (50,191,899–50,191,915 bp) separated by the *Fok*I cleavage zone were used to introduce frameshift deletions in the first coding exon of *Hnrnph1* (exon 4) that resulted in premature stop codons (S8 Fig). Founder #28 contained a 16 bp deletion and Founder #22 contained an 11 bp deletion. (**b):** A PCR amplicon capturing the *Fok*I cleavage zone was digested with BstNI. *Hnrnph1* ^+/+^ mice contained two copies of a functional BstNI restriction site and thus, restriction digest produced a single band containing digested fragments of equal size. *Hnrnph1* ^+/-^ mice were heterozygous for a deletion of the BstNI site and showed both the digested band and a larger, undigested band. Gel band lanes were cropped and re-ordered to present wild-type first (+/+) followed by B6 control, and heterozygous samples (+/-). (**c):** There was a significant upregulation of total *Hnrnph1* transcript levels in *Hnrnph1* ^+/-^ mice as indicated by cDNA amplification using qPCR primers spanning exons 4–5 that hybridized to both genotypes (t_6_ = 5.69; p = 0.0013). (**d):** An upregulation of total *Hnrnph1* transcript levels was also indicated by cDNA amplification using qPCR primers spanning untargeted exons 6–7 (t_6_ = 8.53; p = 0.00014). **(e):** A significant downregulation of the *Hnrnph1* ^+/+^ transcript levels was observed in *Hnrnph1* ^+/-^ mice that was indicated by cDNA amplification using primers spanning exons 4–5, one of which hybridized to the deleted *Hnrnph1* ^+/+^ sequence (t_6_ = 9.45; p = 0.00091; Fig 5e). *p < 0.05. **(f):** In Line #28, there was no effect of genotype on locomotor activity in response to saline (SAL) on Days 1 or 2 (left, middle panels). On Day 3, *Hnrnph1* ^+/-^ mice from Line #28 heterozygotes showed a significant reduction in MA-induced locomotor activity compared to *Hnrnph1* ^+/+^ littermates (right panel). (**g):** In Line #22, there was no effect of genotype on locomotor activity in response to SAL on Days 1 or 2 (left, middle panels). On Day 3, *Hnrnph1* ^+/-^ mice from Line #22 showed significantly reduced MA-induced locomotor activity compared to *Hnrnph1* ^+/+^ littermates. Data are presented as the mean ± S.E.M. * = significant genotype x time interaction followed by unpaired t-tests of individual time bins (p < 0.05; S3 Table; S1 Text; Supplementary Information).

One of the major advantages of genetic analysis in model organisms is the ability to perform experimental manipulations to evaluate observed correlations between genotype and phenotype. We used TALENs to introduce frameshift deletions that resulted in premature stop codons into the first coding exon of each of the two protein coding genes within the 206 kb critical interval–*Hnrnph1* and *Rufy1*. We identified two founders that were heterozygous for 11 bp and 16 bp frameshift deletions in the first coding exon of *Hnrnph1* (*Hnrnph1* ^+/-^; Founders #28 and #22; Figs 5A and S8). We did not observe any off-target deletions in the highly homologous *Hnrnph2* gene nor did we observe compensatory change in striatal *Hnrnph2* expression (S9 Fig).

*Hnrnph1* ^+/-^ mice showed reduced expression of *Hnrnph1*. When we used qPCR primers that hybridized to DNA sequences that were contained in both wild-type (*Hnrnph1* ^+/+^) and *Hnrnph1* ^+/-^ mice, there was a significant upregulation of total *Hnrnph1* transcript levels in *Hnrnph1* ^+/-^ versus *Hnrnph1* ^+/+^ mice (Fig 5C and 5D). However, we also used qPCR primers that overlapped the deleted interval and in this case we observed a significant downregulation of *Hnrnph1* ^+/+^ transcript levels in *Hnrnph1* ^+/-^ mice (Fig 5E). These observations provide functional evidence that the *Hnrnph1* frameshift deletion disrupted gene transcription. Similar to Lines 4, 4a and 4b, *Hnrnph1* ^+/-^ mice from Line #28 and Line #22 that were derived from Founders #28 and #22 both exhibited reduced MA sensitivity (Fig 5F and 5G), thus recapitulating the congenic QTL phenotype. Reduced MA sensitivity was also observed using 30 min behavioral sessions (S10 Fig).

**Fig 6:**
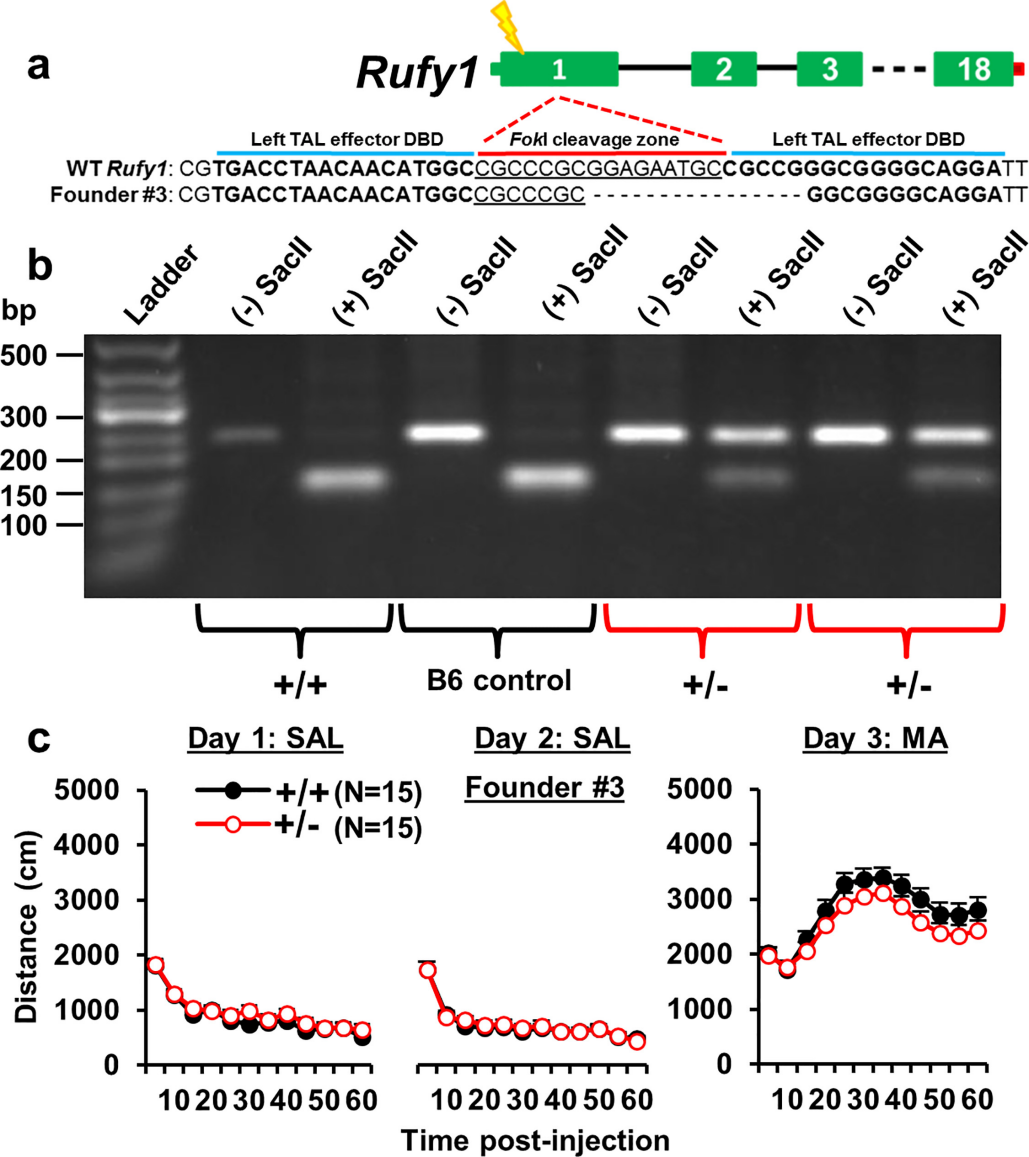
TALENS-targeted frameshift deletion in *Rufy1* ^+/-^ mice. **(a):** A left TALE effector (50,244,600–50,244,616 bp) and a right TALE effector (50,244,569–50,244,585 bp) separated by the *Fok*I cleavage zone were used to introduce a frameshift deletion that resulted in a premature stop codon in the first coding exon of *Rufy1* (see S8 Fig). **(b):** A PCR amplicon was generated that captured the *Fok*I cleavage zone and a single SacII restriction site and was subjected to restriction digest with SacII. *Rufy1* ^+/+^ mice contained the SacII restriction site and thus, showed only a single, smaller band. *Rufy1* ^+/-^ mice showed both the SacII-digested band and a larger, undigested band, indicating the presence of the deletion. **(c):** There was no effect of genotype or genotype x time interaction in *Rufy1* ^+/-^ versus *Rufy1* ^+/+^ mice from Line #3 on Days 1, 2, or 3 (p > 0.05; S3 Table). Data are presented as the mean ± S.E.M.

In contrast to *Hnrnph1* ^+/-^ mice, *Rufy1* ^+/-^ mice carrying a frameshift deletion (S8 Fig) did not exhibit any difference in behavior (Fig 6). To further support the likelihood of reduced neurobehavioral function in *Hnrnph1* ^+/-^ mice, *Hnrnph1* expression is also clearly higher than *Rufy1* in the adult brain (S6 Fig; S11 Fig) [34].

To summarize, we observed a significant reduction in MA sensitivity in *Hnrnph1* ^+/-^ mice, but not *Rufy1* ^+/-^ mice that recapitulated the congenic QTL phenotype, thus identifying *Hnrnph1* as a quantitative trait gene for MA sensitivity.

## Discussion

We used positional cloning and gene targeting to identify *Hnrnph1* as a novel quantitative trait gene for MA sensitivity. First, we identified a broad, time-dependent QTL on chromosome 11 using an F_2_ cross between two inbred strains (Fig 1). We then narrowed a QTL from the initial 40 Mb interval to approximately 10 Mb using interval-specific congenic lines (Figs 2, 3, S2 and S3). Further backcrossing yielded a fortuitous recombination event that narrowed a critical interval to just 206 Kb; this region contained only two protein coding genes: *Hnrnph1* and *Rufy1* (Fig 3E). Striatal transcriptome analysis identified potential neurobiological mechanisms, including a predicted deficit in midbrain dopaminergic neuron development and neurotransmission. The use of GeneNetwork [30] to identify eQTLs associated with our transcriptomic findings provided mechanistic insight, including a *trans*-QTL that maps to *Hnrnph1* that could cause differential expression of *Ipcef1* (Table 2; S6 Table). Finally, we took advantage of the power of mouse genetics to create mice heterozygous for a frameshift deletion in either *Hnrnph1* or *Rufy1*. *Hnrnph1* ^+/-^ mice but not *Rufy1* ^+/-^ mice recapitulated the congenic QTL phenotype, providing direct evidence that *Hnrnph1* is a quantitative trait gene for MA sensitivity (Figs 5 and 6).

QTL mapping studies of rodent behavior have rarely provided strong evidence for causal quantitative trait genes [26, 27, 35]. We began pursuing this QTL more than a decade ago, when the difficulty of such projects was widely underestimated. A key limitation of our initial mapping strategy was the use of an F_2_ cross, in which extensive linkage disequilibrium created large haplotype blocks, resulting in the identification of very broad QTLs. Combining low resolution and high resolution QTL mapping in congenic lines revealed a more complex genetic architecture, indicating that *Hnrnph1* is not the only causal gene within the F_2_ interval that underlies the QTL. Inheritance of two copies of the D2 segment enhanced the heterozygous phenotype in Line 1, yet had no further effect once the size of the segment was reduced following the creation of Line 4 (Fig 2B and 2E). We interpret this observation to suggest that Line 1 contains an additional, recessive QTL within the 35–50 Mb region of Line 3 that could summate with the Line 4 QTL to produce the larger effect size. This 35–50 Mb region could be fine-mapped to the causal genetic factor by introducing additional recombination events into Line 3. This detailed level of insight into the genetic architecture of a single large-effect QTL could only be made possible by employing a sufficiently powered phenotypic analysis of interval-specific congenic lines. Thus, a key to our success in identifying a single gene was the fact that while the QTL originally identified in the F_2_ cross was likely the product of multiple smaller QTLs, we were able to capture one major QTL in Line 4 and in subcongenic lines which appears to correspond to a single quantitative trait gene that we have now identified as *Hnrnph1*.

Transcriptome analysis of Line 4a supports a neurodevelopmental mechanism by which the QTL regulates MA sensitivity. *Nr4a2* (a.k.a. *Nurr1*) was the top downregulated gene and codes for a transcription factor that is crucial for midbrain dopaminergic neuron development, survival, and cellular maintenance of the synthesis, packaging, transport, and reuptake of dopamine [36]. *Nurr1* was a core component of a top-ranked gene network composed of primarily downregulated genes important for neurogenesis, neural differentiation, and synaptogenesis (*Nr4a2 / Nurr1*, *Bdnf*, *Tbr1*, *Neurod6*, *Ets2*, *Malat1*, *Elavl2;* Fig 4). Accordingly, there was a downregulation of striatal signaling pathways, including glutamate (*Slc17a7*, *Slc17a6*, *Gng2*, *and Gria4*), G_αq_ (*Gng2*, *Chrm1*, *Adra1b*, *Adra1d*), and GPCR signaling (*Pde1b*, *Rgs14*, *Chrm1*, *Adra1b*, *Adra1d*) (S8 Table). With regard to G_αq_ signaling, MA acts as a substrate for NET, causing efflux of NE [9] which then binds to α-adrenergic receptors that are coded by *Adra1b* and *Adra1d*. Notably, knockout mice for either of these receptors exhibit reduced amphetamine-induced locomotor activity [37, 38].

Some of the differentially expressed genes in Line 4a were previously associated with variation in amphetamine reward and reinforcement, including *Nr4a2* (*Nurr1*), *Adora2a*, and *Slc17a7* (*Vglut1*) [39]. Furthermore, the top predicted upstream regulator—*Htt* (huntingtin; S7A Fig) is a master regulator of a network of genes in the extended amygdala associated with protracted abstinence from chronic exposure to opioids, cannabinoids, nicotine, and alcohol [40].

Inheritance of the *Hnrnph1* locus caused downregulation of a smaller reverse-transcribed gene located within the middle of *Oprm1* (mu opioid receptor) called *Ipcef1* (p = 0.001; FDR = 12%; S6 Table). We also identified a *trans*-eQTL in *Hnrnph1* that regulates *Ipcef1* expression (Table 2 [30]). *Hnrnph1* was previously shown to regulate the expression *Oprm1* (mu opioid receptor gene) via 5’ UTR-mediated repression [41] and splicing [42]. Furthermore, the human intronic SNP rs9479757 in *OPRM1* was associated with heroin addiction severity and decreased binding affinity of *HNRNPH1*, resulting in exon 2 skipping [43]. Thus, *Hnrnph1* regulation of *Ipcef1* expression could represent an additional mechanism of *Oprm1* regulation [44].

The QTL that contains *Hnrnph1* is predicted to perturb the neural development of the mesocorticolimbic circuitry that mediates MA behavior. *Hnrnph1* (heterogeneous nuclear ribonucleoprotein) codes for an RNA binding protein (RBP) that is highly expressed throughout the brain, including the striatum, cortex, and hippocampus (S11 Fig) [34] and binds to G-rich elements to either enhance or silence splicing [45, 46]. hnRNPs such as *Hnrnph1* form hnRNP-RNA complexes to coordinate splicing of thousands of genes [46]. In addition, *HNRNPH1* regulates 3’ UTR cleavage and polyadenylation [47] and several hnRNPs export mRNAs to neuronal processes to regulate spatiotemporal translation and post-translational modifications [48]. Synaptic activity can increase protein abundance of hnRNPs at the post-synaptic density of primary neurons [49]. The hippocampus contains focal expression of over 15 hnRNPs, including H1 (S11 Fig [34]). Importantly, *Hnrnph1* contains a glycine rich domain that permits nucleocytoplasmic shuttling via transportin 1 [50] and exhibits activity-dependent translocation to the cytoplasm [51]. Several hnRNPs exhibit activity-dependent localization at the synapse [49], suggesting additional neuronal functions of *Hnrnph1* in addition to splicing.

We identified *Hnrnph1* as a quantitative trait gene responsible for MA sensitivity. However, the quantitative trait nucleotide(s) remain obscure. *Hnrnph1* contains 18 genetic variants within the gene, including 15 intronic SNPs, a SNP in the 5’ UTR, a synonymous coding SNP, and a single T insertion in the 3’ UTR (S4 Table [52, 53]) that could cause brain region-specific differential expression of *Hnrnph1* and/or its ability to regulate splicing of its transcriptome-wide targets [46, 47]. We did not observe differential striatal expression of *Hnrnph1* at the gene level or the exon level as a consequence of inheriting the Line 4a QTL (S5 and S6 Figs). Our focus was limited to the striatum which is a behaviorally relevant region [16, 29] that exhibits high *Hnrnph1* expression during early adulthood (S11 Fig). Therefore, the QTL could influence *Hnrnph1* expression at a different time period, in a different, behaviorally relevant brain region, or in a specific subpopulation of cells. Interestingly, striatal microarray datasets in BXD strains indicate an increase in *Hnrnph1* expression from postnatal day 3 to postnatal day 14 as well as a change in the strain rank order of expression [30] which suggests that genotypic differences in *Hnrnph1* expression could depend on the developmental time point. Finally, because excised introns can *trans*-regulate gene expression, an alternative explanation is that excised, SNP-containing introns from *Hnrnph1* can function as polymorphic long noncoding RNAs to perturb their *trans*-regulation of the transcriptome [54].

To our knowledge, there are no GWAS studies reporting genome-wide significant associations of *HNRNPH1* variants with complex diseases or traits (http://www.ebi.ac.uk/gwas/). Interestingly, *HNRNPH1* binding affinity and splicing can be modulated by genome-wide significant SNPs associated with bipolar disorder, major depressive disorder, and schizophrenia, including rs1006737 (*CACNA1C*), rs2251219 (*PBRM1*), and rs1076560 (*DRD2*) [55]. Thus, *HNRNPH1* splicing could profoundly impact the neurobiological mechanisms underlying these disorders. Additionally, *HNRNPH1* and *RBFOX1/2* coordinate splicing [56, 57] and knockdown *RBFOX1* (an autism-associated RBP involved in neural development [58]) in human neural progenitor cells revealed *over* 200 alternatively spliced genes containing *HNRNPH1* binding sites [56] and 524 genes containing binding sites for *ELAVL2*, a neurodevelopmental RBP [59] that was downregulated in Line 4a (Fig 4).

In summary, we identified *Hnrnph1* as a quantitative trait gene for MA sensitivity. This is rarely accomplished in rodent forward genetic studies of behavior and will likely advance our understanding of the neurobiological basis of multiple neuropsychiatric disorders involving monoaminergic dysregulation. Identifying brain region- and cell type-specific splicing targets of *Hnrnph1* could reveal therapeutic targets for these disorders, many of which have been associated with specific gene splicing events [55]. Furthermore, pharmacological perturbation of RBP function could one day serve as an effective therapeutic strategy. Recent findings in models of neurodegenerative disease show that targeting RBP signaling could be a promising treatment approach [60].

## Materials and Methods

### Mice

All procedures in mice were approved by the Boston University and the University of Chicago Institutional Animal Care and Use Committees and were conducted in strict accordance with National Institute of Health guidelines for the care and use of laboratory animals. Colony rooms were maintained on a 12:12 h light–dark cycle (lights on at 0600 h). Mice were housed in same-sex groups of two to five mice per cage with standard laboratory chow and water available *ad libitum*. Age-matched mice were 50–100 days old at the time of testing (0900–1600 h).

### Locomotor activity

For Lines 1–6 and Lines 4a-4h, locomotor activity was assessed in the open field [19]. Briefly, congenics, subcongenics, and wild-type littermates were transported from the vivarium to the adjacent behavioral testing room where they habituated for at least 30 min prior to testing. Mice were then placed into clean holding cages with fresh bedding for approximately five min before receiving an injection of saline on Days 1 and 2 (10 μl/g, i.p) and an injection of methamphetamine on Day 3 (MA; 2 mg/kg, i.p.; Sigma-Aldrich, St. Louis, MO USA). Mice were placed into the center of the open field (37.5 cm x 37.5 cm x 35.7 cm; AccuScan Instruments, Columbus, OH USA) surrounded by a sound attenuating chamber (MedAssociates, St. Albans, VT USA) and the total distance traveled was recorded in six, 5 min bins over 30 min using VersaMax software (AccuScan).

Mice heterozygous for a frameshift deletion in *Hnrnph1* (*Hnrnph1* ^+/-^) or *Rufy1* (*Rufy1* ^+/-^) were engineered (http://www.bumc.bu.edu/transgenic/), bred, and phenotyped at Boston University School of Medicine. Mice were bred and phenotyped in a manner similar to the congenics at the University of Chicago, with the exception that the open field was a smaller size (43.2 cm long x 21.6 cm wide x 43.2 cm tall; Lafayette Instruments, Lafayette, IN USA) and mice were recorded daily for 1 h rather than 30 min to allow a more robust detection of the phenotype. Reduced MA sensitivity was also replicated in *Hnrnph1* ^+/-^ mice using the 30 min protocol (Supplementary Information). Behavior was videotaped using a security camera system (Swann Communications, Melbourne, Australia) and data were collected and analyzed using video tracking (Anymaze, Stoelting, Wood Dale, IL USA).

### Behavioral analysis

Because our primary focus was on MA-induced locomotor activity on Day 3, we first ran a two-way repeated measures ANOVA for Day 3 using genotype and sex as factors and time as the repeated measure. Because sex did not interact with genotype or time for any of the lines on Day 3, we combined sexes for the analysis of Days 1–3 and used repeated measures ANOVA with genotype as the main factor. Main effects of genotype and genotype x time interactions were deconstructed using one-way ANOVAs and Fisher’s post-hoc test of each time bin or t-tests in cases where there were two genotypes. A p-value of less than 0.05 was considered significant.

### QTL analysis of F_2_ mice

B6 x D2-F_2_ mice (N = 676) were generated, maintained, genotyped, and analyzed as previously described [20, 22]. Genome-wide QTL analysis was performed in F_2_ mice using the R package QTLRel that contains a mixed model to account for relatedness among individuals [61]. We recently validated the use of permutation when estimating significance thresholds for mixed models [62]. Sex was included as an interactive covariate. For each analysis, significance thresholds (p < 0.05) were estimated using 1000 permutations. The F_2_ data and R code for are publicly available on github (https:/github.com/wevanjohnson/hnrnph1).

### Generation of congenics and subcongenics

Lines 1 and 6 were obtained from Dr. Aldons Lusis’s laboratory at UCLA (Lines “11P” and “11M” [28]) and had previously been backcrossed to B6 for more than 10 generations. These lines contained homozygous, introgressed regions from D2 on an isogenic B6 background that spanned chromosome 11. Because Lines 1 and 6 contained such large congenic intervals, we first phenotyped non-littermate offspring derived from homozygous congenic breeders versus homozygous B6 wild-type breeders (The Jackson Laboratory, Bar Harbor, ME; Figs 2 and S2) rather than heterozygous-heterozygous breeders to avoid the otherwise high likelihood of introducing unmonitored recombination events. Thus, we ensured that each individual possessed an identical genotype within each congenic line. The same type of control group is typically employed in the initial screen of chromosome substitution strains [19, 63, 64] which are essentially very large congenic lines. We crossed Line 1 to B6 and phenotyped the F_1_ offspring alongside age-matched B6 mice. B6 cohorts were combined into a single group for the combined analysis of all three genotypes for Line 1 (homozygous for B6, homozygous for D2, and heterozygous; Fig 2).

Next, we backcrossed Line 1 heterozygotes to B6 to generate subcongenic Lines 2–5 (Figs 2 and S2). Recombination events were monitored using genomic DNA extracted from tail biopsies and a series of TaqMan SNP markers (Life Technologies; Carlsbad, CA; S1 Table). We then used heterozygous-heterozygous breeding in Lines 2–5 to produce littermates of all three genotypes for simultaneous phenotyping (Figs 2 and S2). Because the QTL in Line 4 represented the smallest congenic region and was dominantly inherited, we backcrossed Line 4 heterozygotes to B6 to generate heterozygotes and wild-type littermates for Lines 4a-4h (Figs 3 and S3). We used additional TaqManSNP markers (Life Technologies) to monitor recombination events and defined the precise congenic boundaries using PCR and Sanger sequencing of SNPs chosen from the Mouse Sanger SNP query database (http://www.sanger.ac.uk/cgi-bin/modelorgs/mousegenomes/snps.pl [52]). Genomic coordinates are based on mm9 (Build 37).

### Test for residual heterozygosity in Lines 4a, 4b, 4c, and 4d

We assayed tail SNP DNA from one heterozygous congenic mouse and one B6 wildtype littermate from Lines 4a-4d (eight mice total) using services provided by the DartMouseSpeed Congenic Core Facility at the Geisel School of Medicine at Dartmouth College (http://dartmouse.org/). A total of 882 informative B6/D2 SNPs were analyzed on the GoldenGate Genotyping Assay (Illumina, Inc., San Diego, CA) using DartMouse’s SNaP-Mapand Map-Synth software to determine the allele at each SNP location. After detecting a single off-target locus on chromosome 3 (rs13477019; 23.7 Mb), we used a custom designed TaqMan SNP marker for rs13477019 (Life Technologies, Carlsbad, CA USA) to confirm the result and to genotype additional samples from Lines 4a-4h for which we had both DNA and behavioral phenotypes. Data from this SNP marker were then used to test for the effect of genotype at the chromosome 3 locus on MA-induced locomotor activity.

### RNA-seq

We harvested and pooled bilateral 2.5 mm diameter punches of the striatum for each individual sample from naïve, congenic mice and B6 wildtype littermates from Line 4a (N = 3 females and 5 males per genotype; 50–70 days old). Total RNA was extracted as previously described [23] and purified using the RNeasy kit (Qiagen, Valencia, CA, USA). RNA was shipped to the University of Chicago Genomics Core Facility where cDNA libraries were prepared for 50 bp single-end reads according to the manufacturer’s instructions using the Illumina TruSeqStranded mRNA LT Kit (Part# RS-122-2101). Purified DNA was captured on an Illumina flow cell for cluster generation and sample libraries were sequenced at eight samples per lane over two lanes (technical replicates) on the Illumina HiSeq 2500 machine according to the manufacturer’s protocols. FASTQ files were quality checked via FASTQC and possessed Phred quality scores > 30 (i.e. less than 0.1% sequencing error). Using the FastX-Trimmer from the FastX-Toolkit, the 51st base was trimmed to enhance read quality and prevent misalignment. FASTQ files were utilized in TopHat [65] to align reads to the reference genome (UCSC Genome Browser). Read counts per gene were quantified using the HTSeq Python package and the R Bioconductor package edgeR was used to analyze differential gene expression. EdgeR models read counts using a negative binomial distribution to account for variability in the number of reads via generalized linear models [66]. “Home cage” was included as a covariate in the statistical model to account for cage effects on gene expression. The p-values obtained for differential expression were then adjusted by applying a false discovery rate (FDR) method to correct for multiple hypothesis testing [67]. The transcriptome dataset and code for RNA-seq analysis are available via NCBI Gene Expression Omnibus (http://www.ncbi.nlm.nih.gov/geo/query/acc.cgi?token=cxkdoeaudvyhlqt&acc=GSE66366).

### Real-time quantitative PCR (qPCR)

Oligo-dT primers were used to synthesize cDNA from total RNA to examine mRNA expression. Primer efficiencies for real-time quantitative PCR (qPCR) experiments were calculated using cycle threshold (*C*_T_) values (SYBR® Green; Life Technologies) derived from five, 10-fold serial cDNA dilutions; efficiencies (E) ranged from 90–100% (R^2^ = 0.99–1). Each sample was run in triplicate and averaged. Differential gene expression was reported as the fold-change in congenic or frameshift-deleted mice relative to B6 wild-type littermates using the 2^-(∆∆C^_T_^)^ method [68].

### Ingenuity pathway analysis (IPA)

We used our differentially expressed gene list from the striatal transcriptome that contained both the log_2_ fold-change and p-values (FDR < 5%) and applied IPA (www.qiagen.com/ingenuity) to identify enriched molecular pathways, functional annotations, gene networks, upstream causes, and predicted neurobiological consequences caused by inheritance of the QTL. IPA utilizes an algorithm that assumes that an increase in the number of molecular interactions indicates an increase in the likelihood of an effect on biological function. IPA uses a manually curated database (IPA Knowledge Base) containing the published literature to extract gene networks containing equally treated edges that directly and indirectly connect biologically related genes (www.qiagen.com/ingenuity). IPA analyses were conducted in February 2015.

#### IPA settings

We considered both direct and indirect relationships that were experimentally observed or moderately-to-highly predicted in all mammalian species, including mouse and rat. We used the “stringent” setting to filter molecules and relationships in tissues and cell lines. With regard to mutations, we considered all functional effects, modes of inheritance, translational impacts, zygosity, wild-type, and unclassified mutation information.

#### Canonical pathways

The ratio of the canonical pathways represents the number of genes in our gene list that overlap with the genes listed in the IPA-generated pathway divided by the total genes within the IPA-generated pathway; thus, a ratio equal to 1 represents perfect overlap. The–log_10_(p-value) for each canonical pathway was derived from the right-tailed Fisher’s exact that measured the degree of overlap between the number of genes identified in our list with the number of genes that comprise the canonical pathway versus the number of genes genome-wide that would be expected to overlap by chance. The p-values were corrected for multiple testing using the Benjamini-Hochberg method [67] and represent the FDR.

#### Diseases, functions, and gene networks

The statistical significance of overlap between our gene list and a particular disease or function was assessed using the p-value derived from a Fisher’s exact test. The predicted activation state was assessed by calculating a z-score that determined the statistical significance of the match between the observed and predicted direction. “Increased” or “decreased” indicates that the Z-score was significant for predicting activated or inhibited state. IPA networks were built based on the degree of connectivity between genes within our gene list, starting with the most connected genes. Genes were added by the IPA algorithm to the network to facilitate connectivity. Networks were limited to a maximum of 35 genes to facilitate interpretability and the ability to generate hypotheses. The Network Score (see S10 Table), a.k.a., the “p score”, represents the–log_10_ (p-value) and represents the probability of finding the observed number of focus genes in a network by chance.

#### Upstream regulator analysis

This analysis identifies causal molecules associated with differential expression using both the significance and the direction of differential expression to specify causal predictions. Several plausible causal networks are constructed and used to calculate an enrichment score and p-value based on overlap between predicted and observed regulator-regulated genes (Fishers exact test). A Z-score is also calculated that determines the degree of match between observed and predicted direction of gene expression (+ or—[69]). “Increased” or “decreased” indicates that the Z-score was significant for predicting activation or inhibition of the regulator.

### GeneNetwork

To identify published *cis*- and *trans*- eQTLs that could explain gene expression differences caused by inheritance of the Line 4a congenic interval, we queried differentially expressed genes (FDR < 20%; 174 genes total; S6 Table) in transcriptome datasets from several brain regions in GeneNetwork [30] involving BXD recombinant inbred strains (recombinant inbred strains derived from B6 and D2 strains). We considered *cis*- and *trans*-QTLs originating from SNPs located within the 50–60 Mb locus and employed an arbitrary cut-off of LRS ≥ 13.8 (LOD ≥ 3). We only included genes where there was an exact match of gene with the LRS location using the appropriate genome build coordinates for each dataset.

### Generation of TALENs-targeted *Hnrnph1* ^+/-^ and *Rufy1* ^+/-^ mice

TALENs vectors encoded either the right or left arm of the TALE effector that targeted the first coding exons of *Hnrnph1* or *Rufy1* (Cellectis Bioresearch, Paris, France). Upon bacterial cloning and purification, TALENs vectors containing a T7 promoter were linearized and used as templates for *in vitro* mRNA synthesis (mMessage mMachine T7 transcription kit; Life Technologies), and purified using MEGAclear transcription clean-up kit (Life Technologies). Each mRNA cocktail was diluted in sterile buffer and injected into B6 single-cell embryos at the BUMC Transgenic Core facility (http://www.bumc.bu.edu/transgenic/). We developed a genotyping assay utilizing native restriction enzyme recognition sites within the TALENs *Fok*I cleavage domain. Genomic DNA was extracted from mouse tail biopsies and PCR-amplified with primers targeting100 base pairs upstream and downstream of the TALENs binding domain. Amplicons were then exposed to restriction digest overnight, run on a 2% agarose Ethidium Bromide Tris-Borate-EDTA gel, and imaged with ultraviolet light. TALENs-targeted deletions were identified by the presence of undigested bands caused by a loss of the restriction site. To confirm base pair deletions in our founder lines, undigested restriction enzyme-exposed PCR amplicon bands were excised, gel-purified, and vector-ligated overnight at 4°C using the pGEM T-easy Vector Systems (Promega). The ligation reaction was transformed into MAX Efficiency DH5α Competent Cells (Invitrogen) and plated onto Ampicillin-IPTG/X-Gal LB agarose plates for blue-white selection. Following overnight incubation at 37°C, white colonies were picked, cultured in ampicillin-enriched LB medium, and amplified. The PCR product was purified using the QIAprep Miniprep kit (QIAGEN). We then sequenced the vectors for the deletions using the pGEM T7 site upstream of the insert.

### Genotyping of TALENs-targeted *Hnrnph1* ^+/-^ and *Rufy1* ^+/-^ mice

An *Hnrnph1* forward primer (GTTTTCTCAGACGCGTTCCT) and reverse primer (ACTGACAACTCCCGCCTCA) were designed to target upstream and downstream of the TALENs binding domain in exon 4 of *Hnrnph1*. Genomic DNA was used to amplify a 204 bp PCR product using DreamTaq Green PCR Mastermix (ThermoScientific). PCR products were treated with the BstNI restriction enzyme (New England Biolabs) or a control enzyme-free buffer solution and incubated overnight at 60°C to ensure complete digestion. Enzyme-treated PCR products and untreated controls were resolved in 2% agarose gel electrophoresis with 0.5 μg/mL ethidium bromide to visualize under UV light. There were two BstNI restriction sites within the *Hnrnph1* amplicon that were located proximal and distal to the TALENs *Fok*I cleavage zone. Mice heterozygous for the *Hnrnph1* deletion showed two bands on the gel, while B6 controls showed a single band.

Similar to *Hnrnph1*, a *Rufy1* forward primer (AATCGTACTTTCCCGAATGC) and reverse primer (GGACTCTAGGCCTGCTTGG) targeted upstream and downstream of the TALENs binding domain in the first coding exon (exon 1). The 230 bp PCR amplicon contained a SacII restriction site that was deleted in *Rufy1* ^+/-^ mice. Thus, *Rufy1* ^+/+^ mice showed a single, smaller digested band whereas *Rufy1* ^+/-^ mice showed both the digested band as well as a larger, undigested band.

### Assessment of potential off-target deletion of *Hnrnph2* in *Hnrnph1*-targeted mice

To assess off-target activity in *Hnrnph1*-targeted mice, we used the UCSC genome browser to BLAT the TALENs binding domains and identified a single homologous region located within the first coding exon of *Hnrnph2*. We used the same PCR- and gel-based assay to test for the deletion in *Hnrnph2* with the exception that we used forward (GCCACCAAGAGTCCATCAGT) and reverse primers (AATGCTTCACCACTCGGTCT) that uniquely amplified a homologous 197 bp sequence within *Hnrnph2* that contained a single Bstn1 restriction site. Digestion at the Bstn1 site produced an 81 bp band and a 115 bp band.

## Supporting Information

S1 Text Statistical analyses are provided for Lines 1–6 and Lines 4a-4h. Additional details are also provided for assessment of residual heterozygosity and statistical analyses for *Hnrnph1* ^+/-^ and *Rufy1* ^+/-^ mice.
(DOCX)

S1 Table SNPs that define Lines 1 through 6. B6 = homozygous for C57BL/6J; D2 = homozygous for DBA/2J; H = heterozygous; ARRAY = SNP array-based genotyping; TAQMAN = custom-designed fluorescent SNP genotping; SEQ = Sanger sequencing-based genotyping; ND = not determined
(XLSX)

S2 Table SNPs that define Lines 4a-4h. SNP ID, chromosome 11 location (mm9) method of genotyping, and genotypes are listed. B6 = homozygous for C57BL/6J; D2 = homozygous for DBA/2J; H = heterozygous; ARRAY = SNP array-based genotyping; SEQ/TAQ = SNPs were both Sanger-sequenced and genotyped using custom-designed Taqman fluorescent SNP genotyping; SEQ = Sanger sequencing-based genotyping; NI = non-informative; ND = not determined. Red-filled cells denote the critical interval spanning 50,185,512–50,391,845 bp.
(XLSX)

S3 Table ANOVA tables for congenic lines and TALENs-targeted lines. F statistics and p-values are listed for Days 1, 2, and 3 for the effect of genotype (Geno) and Geno x Time interactions as well as significant time bins.
(XLSX)

S4 Table Genetic variants between B6 and D2 within critical interval. Data (mm9) were obtained from the Sanger mouse query tool containing genetic variants (http://www.sanger.ac.uk/resources/mouse/genomes/).
(XLSX)

S5 Table Residual heterozygosity in Lines 4a through 4d. One mouse for each genotype (B6 = homozygous for B6 allele; H = heterozygous for B6 and D2 alleles) from each of the four congenic lines (Lines 4a, 4b, 4c, and 4d) were genotyped using services provided by DartMouse. The SNP ID, chromosome (Chr.), physical position (Build 34), and genotype are listed. Each SNP allele is represented by either “A” or “B” and the reference allele (“JAX B6”) which could be either AA or BB. NC = no call.
(XLSX)

S6 Table Differentially expressed genes in the striatum of Line 4a. Gene ID, gene name, physical position and build, log2 fold-change, fold-change (FC), P-value, and Q-value are listed in order of ascending p-value.
(XLSX)

S7 Table Primer sequences used for qPCR. Genes, targeted exons, forward and reverse sequences, and amplicon size (bp) are listed.
(XLSX)

S8 Table Canonical pathways in IPA. The pathway, -logP, ratio, z-score, and genes (“Molecules”) identified from our list are shown. The top 20 annotations are listed.
(XLSX)

S9 Table Diseases and functions annotations. The z-score indicates the degree of match between the observed and predicted “Increased” or “decreased” denotes those Z-score that were significant.disease or function. The top 20 annotations are shown.
(XLSX)

S10 Table Top IPA networks containing disease and functions annotations. Score [p score; -log10(p-value], number of focus genes identified from our gene list, and names of diseases and function associated with each network are shown.
(XLSX)

S1 Fig Distal QTL on chromosome 11 (90 Mb) for days 1 and 2 that increased locomotor activity in response to saline (D2 > B6). We previously published a genome-wide significant QTL on chromosome 11 for Day 1 and Day 2 from this B6 x D2-F_2_ dataset that was significant from 0–15 min and from 15–30 min ^20^. Here, we report the LOD scores from the same dataset in six, 5-min time bins over 30 min. **(a, b):** QTL plots are shown for the time bins on Day 1 (saline; SAL, i.p.) and Day 2 (SAL, i.p.). The x-axis represents the physical location of the marker (Mb). The y-axis represents the LOD score. The dashed, horizontal line represents the genome-wide significance threshold derived from 1,000 permutations. The dashed QTL trace indicates the time bin containing the most significant LOD score for each day. The peak LOD was observed at approximately 90 Mb; this same QTL was also present on Day 3 at the first 5-min bin prior to the behavioral onset of MA (Fig 1A). **(c, d):** Effect plot of the marker with the most significant LOD scores is shown for Day 1 and Day 2 in 5-min time bins. Data are sorted by genotype at the marker rs3710148 (96.4 Mb) for each time bin. The time bin with the most significant LOD score is circled. B6 = homozygous for the B6 allele (black circles); H = heterozygous (open triangles); D2 = homozygous for the D2 allele (colored squares). Data are presented as the mean ± S.E.M.
(TIF)

S2 Fig MA sensitivity in Line 5 and Line 6. Lines 5 and 6 possessed chromosome 11 intervals from the D2 strain on an isogenic B6 background (see Fig 2A). The SNPs used to define the intervals in Lines 5 and 6 are listed in S1 Table. **(a, b):** The three columns represent the locomotor phenotypes for Days 1, 2, and 3 for Line 5 and Line 6. Sample sizes (N) are listed for each genotype. Data are presented as the mean ± S.E.M. Statistical analyses are included in S3 Table.
(TIF)

S3 Fig MA sensitivity in Lines 4d-4h. Lines 4d-4h were derived from Line 4 and possessed heterozygous intervals from the D2 strain on an isogenic B6 background (see Fig 3A). The SNPs used to define Lines 4d-h are listed in S2 Table. **(a-e):** The three columns represent the locomotor phenotypes for Days 1, 2, and 3. The five rows (a-e) represent the phenotypes for Lines 4d-4h, respectively. Sample sizes (N) are listed for each genotype. There was no effect of genotype or genotype x time interaction on MA-induced locomotor activity for any of these lines (see S3 Table). Data are presented as the mean ± S.E.M.
(TIF)

S4 Fig Physical map of the 882 genome-wide informative markers used to ascertain residual heterozygosity in Lines 4a-4d. **(a):** The sample that is shown is a Line 4a heterozygous mouse that was genotyped with the GoldenGate SNP microarray (services and figure were provided by DartMouse; http://dartmouse.org/). As expected, this mouse was heterozygous for B6 and D2 alleles at all three SNP markers within the Line 4a congenic region on chromosome 11 (purple, horizontal ticks). Additionally, this mouse was heterozygous at a marker located on chromosome 3 (rs13477019; 23.7 Mb; purple, horizontal tick). This region of residual heterozygosity also segregated in Lines 4b-4h. All other markers were genotyped as homozygous for the B6 allele (green, horizontal ticks). S5 Table lists the complete set of SNPs and genotypes for the eight samples tested on the array. (**b):** When sorting by genotype on chromosome 3 (rs13477019) in 115 mice from Lines 4a-4h for which we had both genotypic and phenotypic information available, there was no effect of genotype (F_2,112_ < 1) or genotype x time interaction with regard to MA sensitivity (F_5,560_ < 1). Data are presented as the mean ± S.E.M.
(TIF)

S5 Fig qPCR results for *Hnrnph1* and *Rufy1* expression in the striatum in Line 4a. **(a):** Heterozygous (H) mice (N = 8) showed significantly reduced *Nurr1* expression relative to B6 (N = 8; t_14_ = 2.18; p = 0.047). **(b, c):** There was no significant difference in expression of *Hnrnph1* (exons 12–13; t_29_ < 1) or *Rufy1* (exons 16–17; t_29_ = 1.51; p = 0.14) in B6 (N = 14) versus H (N = 17) mice. Data are presented as the mean ± S.E.M. Primer sequences are listed in S7 Table.
(TIF)

S6 Fig Exon-level read counts for *Hnrnph1* and *Rufy1* in Line 4a using Integrated Genome Browser. **(a, b):** The x-axis represents the physical location (bp) of the annotated exons (vertical lines, UCSC Genome Browser; mm9) on chromosome 11 for *Hnrnph1* and *Rufy1*. The y-axis represents the summed read counts (y-axis) across all 8 samples for each genotype (B6, H). Note that different scales are used on the y-axis for *Hnrnph1* (the more highly expressed gene; 0–2500 reads) versus *Rufy1* (0–300 reads).
(TIF)

S7 Fig *Htt* and *Creb1* are the top two IPA upstream regulators of the striatal transcriptome in Line 4a. Arrows pointing toward genes indicate predicted activation; horizontal, perpendicular lines indicate predicted inhibition. Green and red colors indicate downregulated or upregulated genes in our dataset. Purple circles denote genes that overlap between *Htt* (a) and *Creb1* (b). The legend on the right hand side denotes the biological classification for each gene contained in the regulator diagrams.
(TIF)

S8 Fig TALENs-targeted *Hnrnph1* and *Rufy1* deletions produce frameshift mutations that result in premature stop codons. We used the ExPASy Translate Tool (http://web.expasy.org/translate/) to input wild-type and deleted cDNA sequences to obtain protein sequences. **(a-c):** Amino acid sequence is shown for *Hnrnph1* ^+/+^ mice and *Hnrnph1* ^+/-^ founders. **(d, e):** Amino acid sequence is shown for *Rufy1* ^+/+^ and *Rufy1* ^+/-^ founders. Methionine (Met) is shown in green. A red “Stop” denotes a stop codon.
(TIF)

S9 Fig No off-target deletions in the highly homologous *Hnrnph2* gene and no compensatory change in *Hnrnph2* expression in *Hnrnph1* ^+/-^ mice. **(a):** A 197 bp PCR amplicon was generated using primers specific for exon 4 of *Hnrnph2* and contained the same homologous BstNI cut site as exon 4 in *Hnrnph1* (Fig 5). *Hnrnph1* ^+/+^ mice and *Hnrnph1* ^+/-^ founder mice (#28 and #22) that were heterozygous for an *Hnrnph1* frameshift deletion all showed two bands following restriction digest, indicating that there was no deletion of the restriction site in *Hnrnph2*. **(b):** There was no compensatory change in *Hnrnph2* expression in Line #28 when comparing *Hnrnph1* ^+/-^ (N = 4) versus *Hnrnph1* ^+/+^ (N = 4) mice (t_6_ < 1). Data are presented as the mean ± S.E.M.
(TIF)

S10 Fig Reduced MA sensitivity in TALENs-targeted *Hnrnph1* ^+/-^ mice (Founder #28 Line) following 30 min training sessions. **(a):** For Day 1, there was no effect of genotype (F_1,32_ < 1) nor any interaction with time (F_5,160_ <1). **(b):** For Day 2, there was no effect of genotype (F_1,32_ = 3.79; p = 0.06) but there was a significant genotype x time interaction (F_5,160_ = 3.66; p = 0.0037 that was explained by *Hnrnph 1* ^+/-^ mice showing significantly greater locomotor activity than *Hnrnph1* ^+/+^ mice at the 5-min and 10-min time bins (t_32_ = 2.53, 2.42; p = 0.017, 0.021). **(c):** For Day 3, there was an effect of genotype (F_1,32_ = 5.37; p = 0.027) but no significant genotype x time interaction (F_5,160_ = 2.04; p = 0.076). *Hnrnph1* ^+/-^ mice showed significantly less MA-induced locomotor activity than *Hnrnph1* ^+/+^ mice at 25 and 30 min (t_32_ = 2.07, 3.03; p = 0.046, 0.0048). Data are presented as the mean ± S.E.M. *p < 0.05.
(TIF)

S11 Fig Mid-sagittal, *in situ* hybridization sections for *Hnrnph1* and *Rufy1*. *In situ* hybridization staining of mid-sagittal sections are shown for *Hnrnph1* (panel a) and *Rufy1* (panel b) and were obtained from the Allen Institute for Brain Science (http://www.brain-map.org/ ^4^). *Hnrnph1* clearly shows higher expression than *Rufy1* which can also evident in the number of read counts in our dataset (see also S6 Fig).
(TIF)
