# Supplementary material for: Hnrnph1 Is A Quantitative Trait Gene for Methamphetamine Sensitivity
Source: PLoS Genet. 2015 Dec 10;11(12):e1005713. doi: 10.1371/journal.pgen.1005713 (PMC4675533; doi:10.1371/journal.pgen.1005713)
Supplement: S1 Text — Additional details are also provided for assessment of residual heterozygosity and statistical analyses for Hnrnph1 +/- and Rufy1 +/- mice. (DOCX) [file pgen.1005713.s001.docx]

**Results**

**Lines 1-6 and Lines 4a-4h**

Statistical results that include main effects of genotype, time, and genotype x time interactions are provided in S3 Table. Here, we report significant genotype x time interactions as well as follow-up one-way ANOVAs and Fisher’s LSD post-hoc test or unpaired t-tests for each time bin to deconstruct these interactions.

**Line 1.** For Day 1, repeated measures ANOVA indicated a significant genotype x time interaction (F_10,585_ = 5.62; p < 0.0001) that was explained by an effect of genotype at all six time bins (F_2,117_ = 33.67, 27.82, 25.46, 37.41, 15.44, and 17.36; p < 0.0001) and by an additive mode of inheritance. Heterozygous (H) mice showed an intermediate phenotype (Figure 2b, left panel, “$”). For Day 2, the genotype x time interaction (F_10,585_ = 6.67; p < 0.0001) was explained by an effect of genotype at all six time bins (F_2,117_ = 33.31, 10.07, 17.37, 18.19, 9.41, and 6.51; p < 0.01) and by time-dependent dominant and additive modes of inheritance (Figure 2b, middle panel, “*” and “$”). For Day 3, the genotype x time interaction (F_10,585_ = 18.06; p < 0.0001) was explained by an effect of genotype at all six time bins (F_2,117_ = 64.49, 51.0, 38.30, 45.14, 47.15, 45.40; p < 0.0001) and by an additive mode of inheritance (Figure 2b, right panel, “$”).

**Line 3.** For Day 1, repeated measures ANOVA indicated a significant genotype x time interaction (F_10,285_ = 3.13; p = 0.0008) that was explained by an effect of genotype at 5, 10, and 30 min (F_2,57_ = 7.85, 3.47, 5.69; p = 0.01, 0.038, 0.0056) and by a dominant mode of inheritance (Fig. 2d, left panel, “*”). For Day 3, the genotype x time interaction was explained by an effect of genotype at 25 and 30 min (F_2,57_ = 4.09, 5.74; p = 0.022, 0.0054) and by a recessive mode of inheritance whereby two copies of the D2 allele (“D2”) were required for phenotypic expression (Fig. 2d, right panel, “#”).

**Line 4.** For Day 3, the genotype x time interaction (F_10,505_ = 1.85; p = 0.049) was explained by an effect of genotype at 5, 10, 20, and 25 min (F_2,101_ = 3.85, 3.15, 3.34, 3.5; p = 0.024, 0.047, 0.040, 0.034) and by a dominant mode of inheritance (Fig. 2e; right panel, “*”) whereby H and D2 mice demonstrated comparable phenotypic expression.

**Lines 2, 5, and 6.** There were no genotype x time interactions for Days 1, 2, or 3 in Lines 2, 5, and 6 (p > 0.05; Fig. 2c; S2 Fig.; S3 Table).

**Line 4a.** For Day 3, the genotype x time interaction (F_5,230_ = 4.92; p = 0.0003) was explained by H mice showing significantly less activity than B6 mice at 15, 20, 25, and 30 min (t_46_ = 2.11, 2.17, 2.16, 2.54; p = 0.04, 0.035, 0.036, 0.015; Fig. 3b, right panel, “*”).

**Line 4b.** For Day 3, the genotype x time interaction (F_5,255_ = 3.72; p = 0.0029) was explained by H mice showing significantly less activity than B6 mice at 10, 15, 20, 25, and 30 min (t_51_ = 2.43, 2.8, 2.70, 2.64, 2.24; p = 0.016, 0.0072, 0.0094, 0.01, 0.029; Fig. 3c, right column, “*”).

**Power analysis of Line 4b.** After replicating the Line 4 QTL in 4a and 4b, we further dissected this locus (Lines 4c-4h). We first determined the sample size required to detect the effect of Line 4b on Day 3 (25 min; peak QTL effect – Figure 1a). We used the means and standard deviations of B6 and H groups in G*Power3 (<http://www.psycho.uni-duesseldorf.de/aap/projects/gpower/>) and calculated an effect size of r = 0.34 (Cohen’s *d* = 0.72). A sample size of N = 25 was required to achieve 80% statistical power with a Type I error rate of 5%. Accordingly, we employed a minimum of N = 25 per genotype in Lines 4c and 4d (both derived from Line 4b).

**Lines 4d, e, f, g, and h.** For Days 1-3, there was no significant genotype x time interaction (p > 0.05; S2 Fig.; S3 Table).

**Residual heterozygosity in Line 4 subcongenics**

Congenic mice were homozygous for the B6 background strain for all SNPs tested on the array with the exception of a single SNP on chromosome 3 (rs13477019; 23,723,842 bp) that segregated in both wild-type and heterozygous congenic genotypes of Lines 4a-4d (S5 Table; S4a Fig.). All samples that were ascertained on the array were homozygous for B6 at the two flanking array markers located at 21.22 and 26.41 Mb on chromosome 3 (S5 Table). Furthermore, in genotyping 115 mice from Lines 4a-4h in cases where we had both DNA and phenotype information available, there was no effect of genotype or genotype x time interaction at the chromosome 3 locus on MA-induced locomotor activity (S4b Fig.). Finally, we previously did not observe any QTL on chromosome 3 for MA-induced locomotor activity in B6 x D2-F_2_ or -F_8_ mice ^3^. Taken together, these results indicate that the differences observed in Lines 4a and 4b are not due to residual heterozygosity.

**Recapitulation of the congenic phenotype in *Hnrnph1* ^+/-^ mice, but not *Rufy1* ^+/-^ mice**

For Days 1 and 2, there was no effect of genotype or interaction with time in Line #28 or Line #22 (p > 0.05; Table S3). On Day 3 in Line #28, repeated measures ANOVA indicated a significant effect of genotype (F_1,38_ = 6.78; p = 0.013) and a genotype x time interaction (F_11,418_ = 2.36; p=0.0079) was explained by *Hnrnph1***^+/-^** mice showing significantly less MA-induced locomotor activity than WT mice at 10, 15, 20, 25, 30, and 35 min (t_38_ = 3.21, 3.82, 3.61, 3.19, 2.25, 2.64; p = 0.0027, 0.00048, 0.00088, 0.0029, 0.030, 0.012; Fig. 5f). We obtained nearly identical results for Line #22. On Day 3, there was a significant effect of genotype (F_1,43_ = 8.17; p = 0.0065) and a genotype x time interaction (F_11,473_ = 3.76; p = 3.57 x 10^-5^) was explained by *Hnrnph1***^+/-^**  mice showing a significant decrease in MA-induced locomotor activity at 10, 15, 20, 25, 30, 35, 40, 45, 50, 55, and 60 min (t_43_ = 2.65, 2.79, 2.49, 2.24, 2.39, 2.52, 2.37, 2.28; p = 0.033, 0.014, 0.012, 0.0081, 0.0044, 0.0023, 0.0047, 0.0081; Fig. 5g).
