# Supplementary figures and images for: Hnrnph1 Is A Quantitative Trait Gene for Methamphetamine Sensitivity
Source: PLoS Genet. 2015 Dec 10;11(12):e1005713. doi: 10.1371/journal.pgen.1005713 (PMC4675533; doi:10.1371/journal.pgen.1005713)

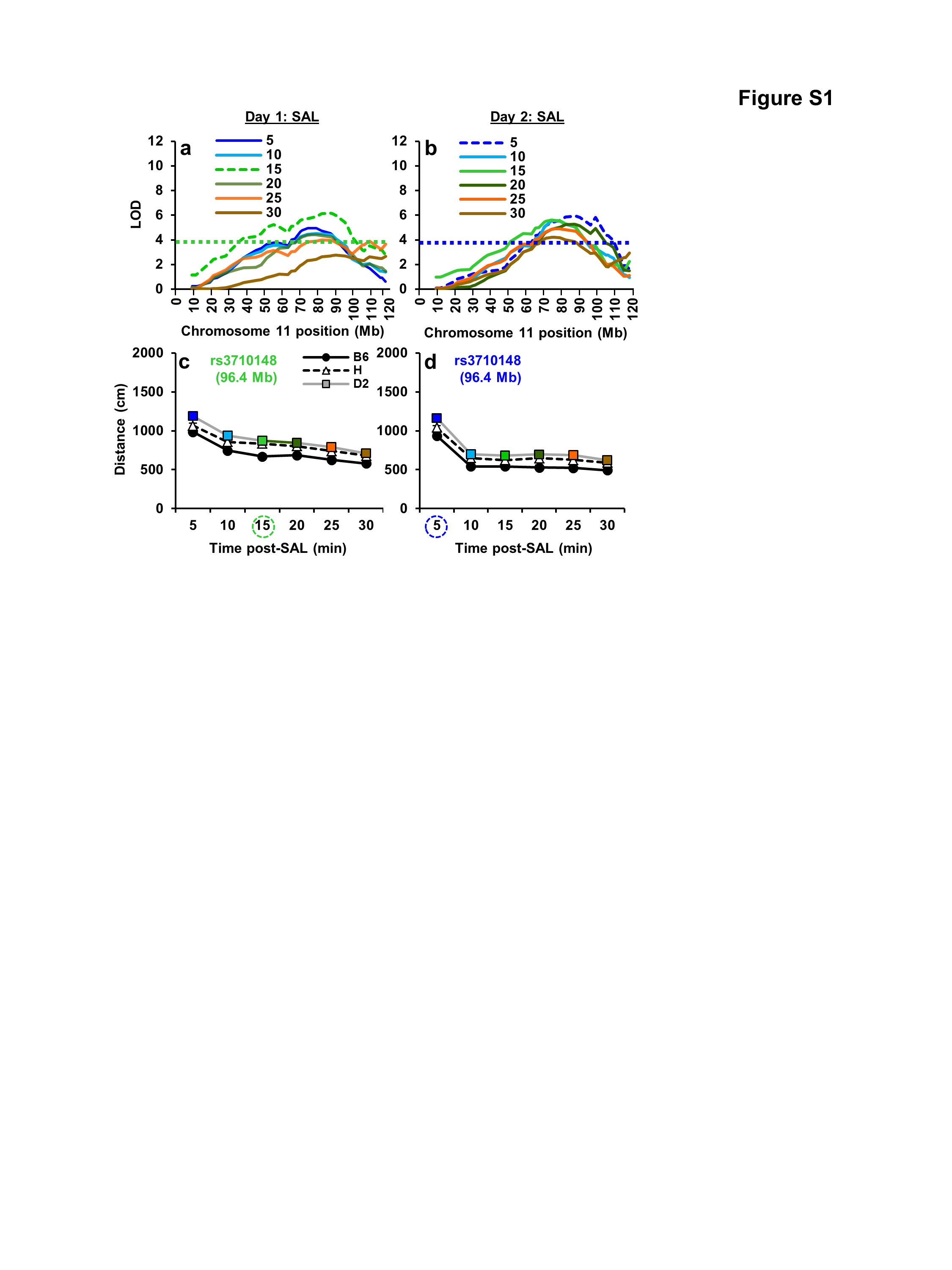

Supplement: S1 Fig — We previously published a genome-wide significant QTL on chromosome 11 for Day 1 and Day 2 from this B6 x D2-F2 dataset that was significant from 0–15 min and from 15–30 min 20. Here, we report the LOD scores from the same dataset in six, 5-min time bins over 30 min. (a, b): QTL plots are shown for the time bins on Day 1 (saline; SAL, i.p.) and Day 2 (SAL, i.p.). The x-axis represents the physical location of the marker (Mb). The y-axis represents the LOD score. The dashed, horizontal line represents the genome-wide significance threshold derived from 1,000 permutations. The dashed QTL trace indicates the time bin containing the most significant LOD score for each day. The peak LOD was observed at approximately 90 Mb; this same QTL was also present on Day 3 at the first 5-min bin prior to the behavioral onset of MA (Fig 1A). (c, d): Effect plot of the marker with the most significant LOD scores is shown for Day 1 and Day 2 in 5-min time bins. Data are sorted by genotype at the marker rs3710148 (96.4 Mb) for each time bin. The time bin with the most significant LOD score is circled. B6 = homozygous for the B6 allele (black circles); H = heterozygous (open triangles); D2 = homozygous for the D2 allele (colored squares). Data are presented as the mean ± S.E.M. (TIF) [file pgen.1005713.s012.tif]

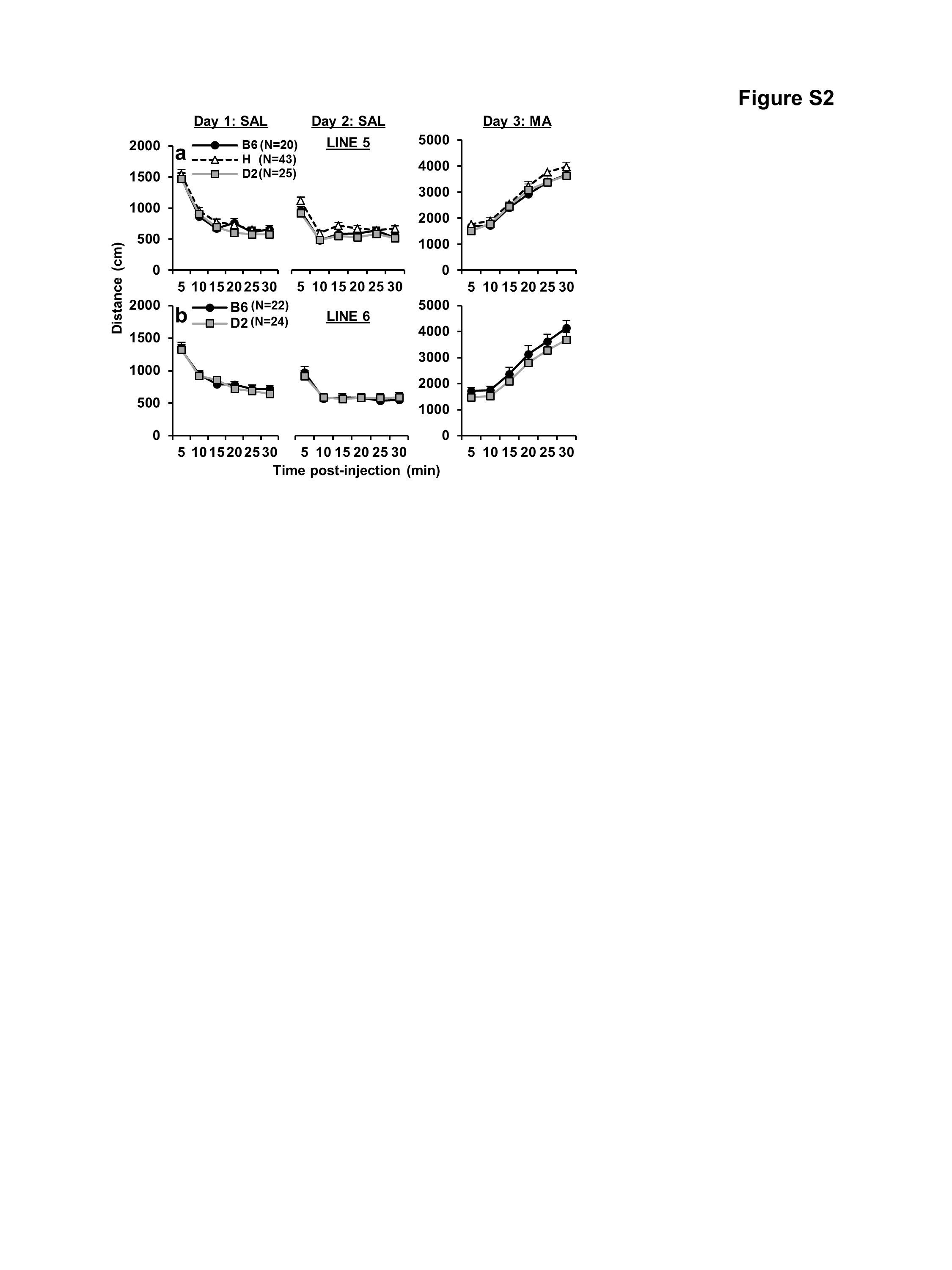

Supplement: S2 Fig — Lines 5 and 6 possessed chromosome 11 intervals from the D2 strain on an isogenic B6 background (see Fig 2A). The SNPs used to define the intervals in Lines 5 and 6 are listed in S1 Table. (a, b): The three columns represent the locomotor phenotypes for Days 1, 2, and 3 for Line 5 and Line 6. Sample sizes (N) are listed for each genotype. Data are presented as the mean ± S.E.M. Statistical analyses are included in S3 Table. (TIF) [file pgen.1005713.s013.tif]

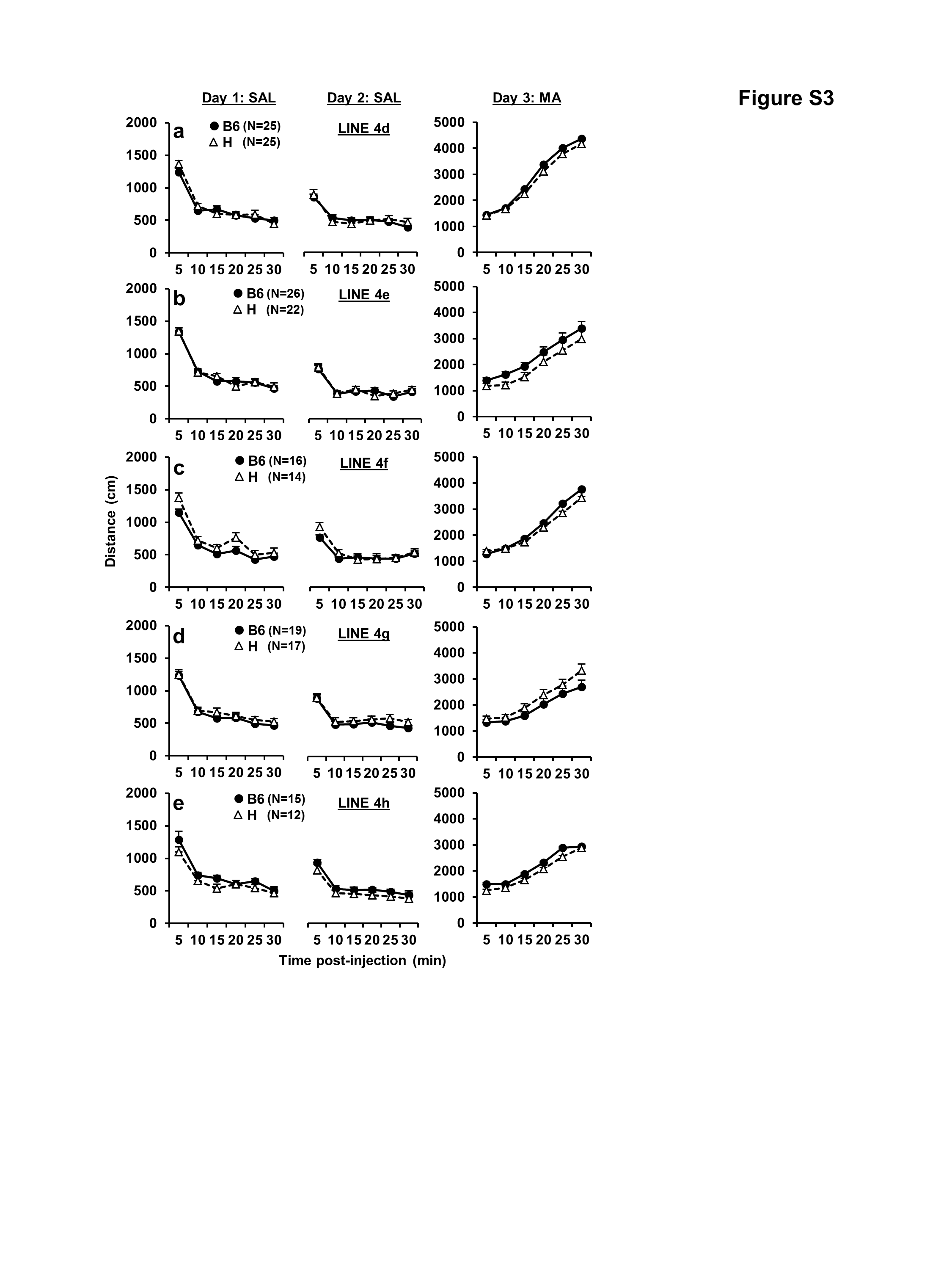

Supplement: S3 Fig — Lines 4d-4h were derived from Line 4 and possessed heterozygous intervals from the D2 strain on an isogenic B6 background (see Fig 3A). The SNPs used to define Lines 4d-h are listed in S2 Table. (a-e): The three columns represent the locomotor phenotypes for Days 1, 2, and 3. The five rows (a-e) represent the phenotypes for Lines 4d-4h, respectively. Sample sizes (N) are listed for each genotype. There was no effect of genotype or genotype x time interaction on MA-induced locomotor activity for any of these lines (see S3 Table). Data are presented as the mean ± S.E.M. (TIF) [file pgen.1005713.s014.tif]

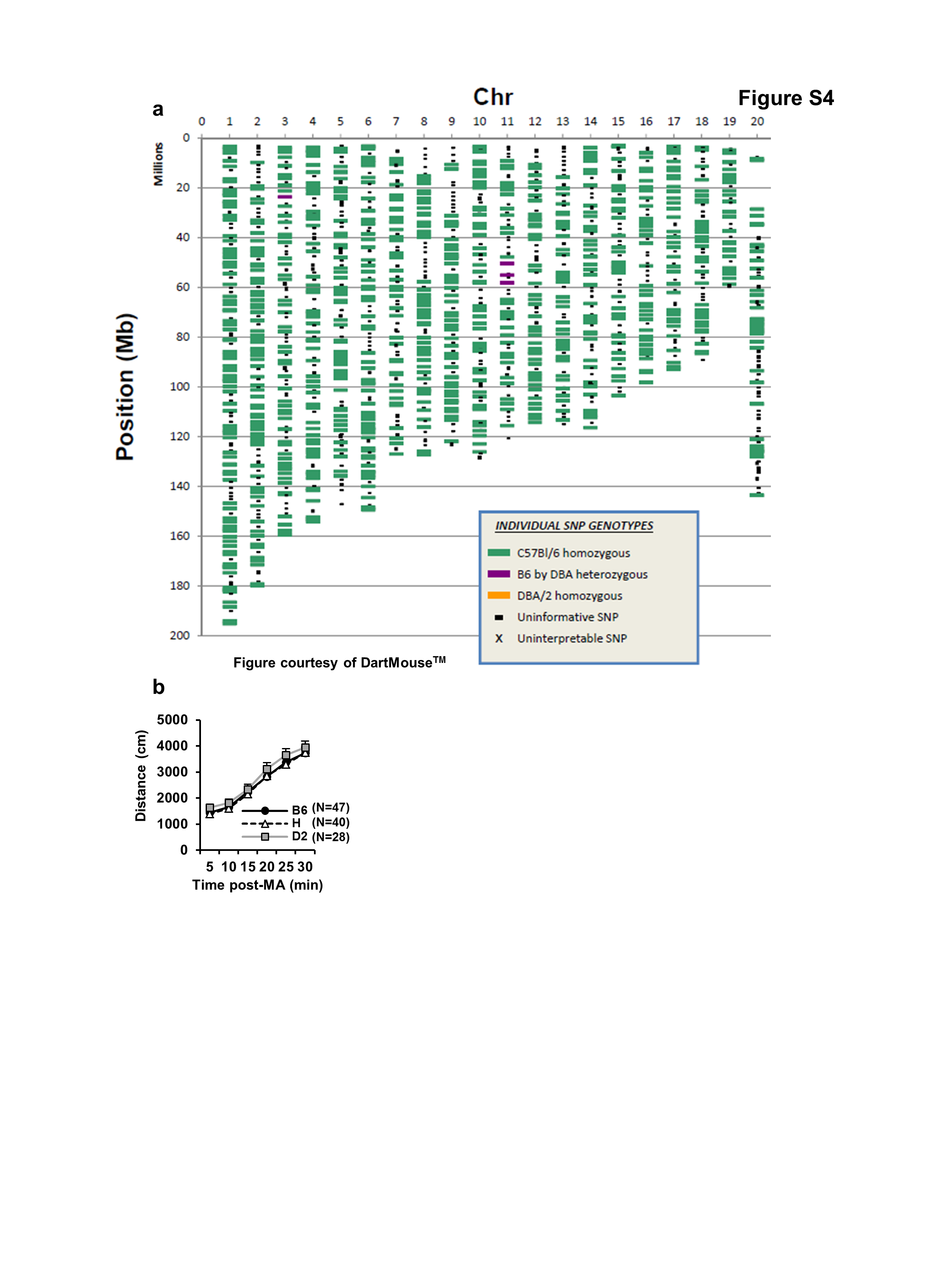

Supplement: S4 Fig — (a): The sample that is shown is a Line 4a heterozygous mouse that was genotyped with the GoldenGate SNP microarray (services and figure were provided by DartMouse; http://dartmouse.org/). As expected, this mouse was heterozygous for B6 and D2 alleles at all three SNP markers within the Line 4a congenic region on chromosome 11 (purple, horizontal ticks). Additionally, this mouse was heterozygous at a marker located on chromosome 3 (rs13477019; 23.7 Mb; purple, horizontal tick). This region of residual heterozygosity also segregated in Lines 4b-4h. All other markers were genotyped as homozygous for the B6 allele (green, horizontal ticks). S5 Table lists the complete set of SNPs and genotypes for the eight samples tested on the array. (b): When sorting by genotype on chromosome 3 (rs13477019) in 115 mice from Lines 4a-4h for which we had both genotypic and phenotypic information available, there was no effect of genotype (F2,112 < 1) or genotype x time interaction with regard to MA sensitivity (F5,560 < 1). Data are presented as the mean ± S.E.M. (TIF) [file pgen.1005713.s015.tif]

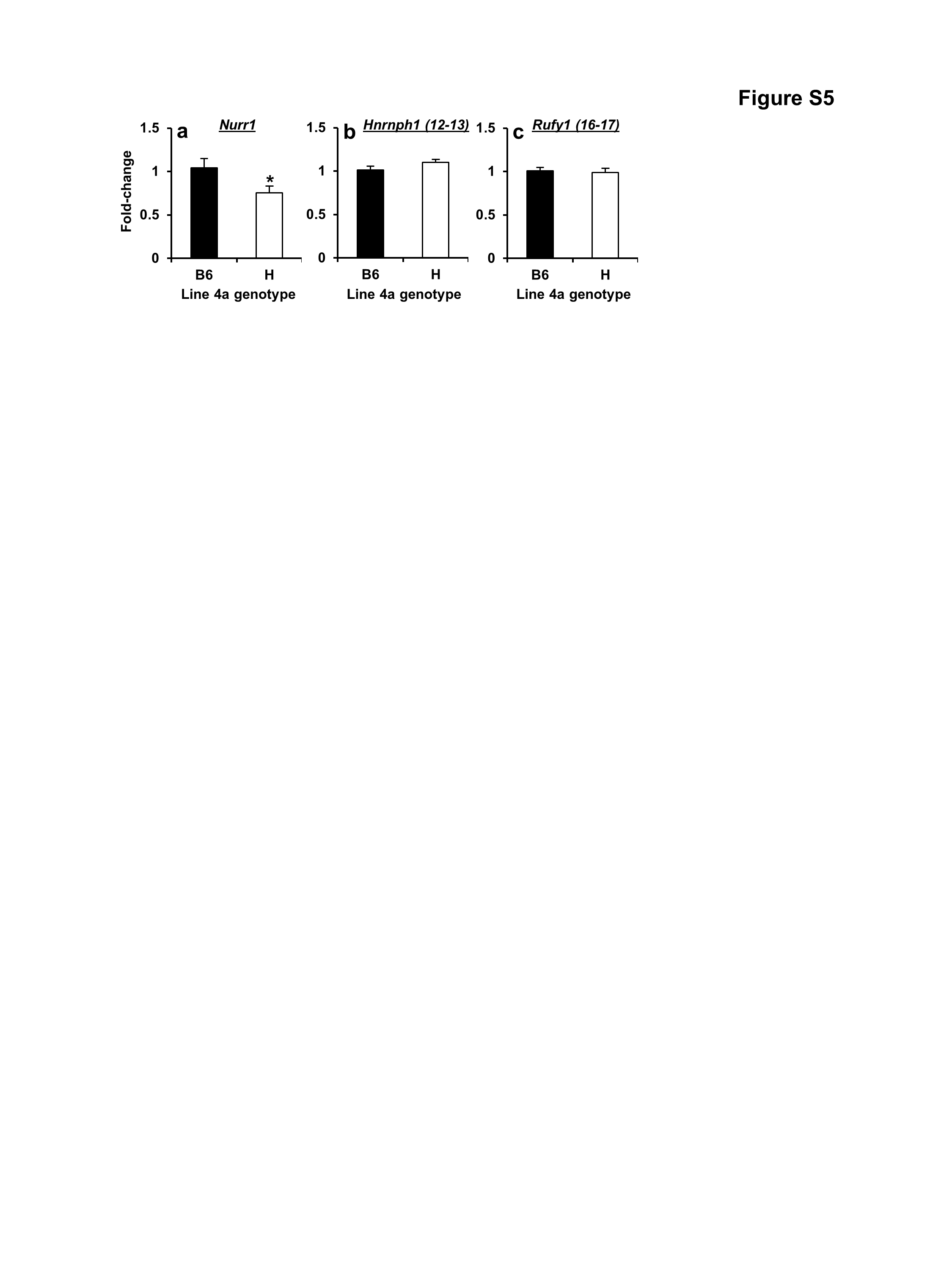

Supplement: S5 Fig — (a): Heterozygous (H) mice (N = 8) showed significantly reduced Nurr1 expression relative to B6 (N = 8; t14 = 2.18; p = 0.047). (b, c): There was no significant difference in expression of Hnrnph1 (exons 12–13; t29 < 1) or Rufy1 (exons 16–17; t29 = 1.51; p = 0.14) in B6 (N = 14) versus H (N = 17) mice. Data are presented as the mean ± S.E.M. Primer sequences are listed in S7 Table. (TIF) [file pgen.1005713.s016.tif]

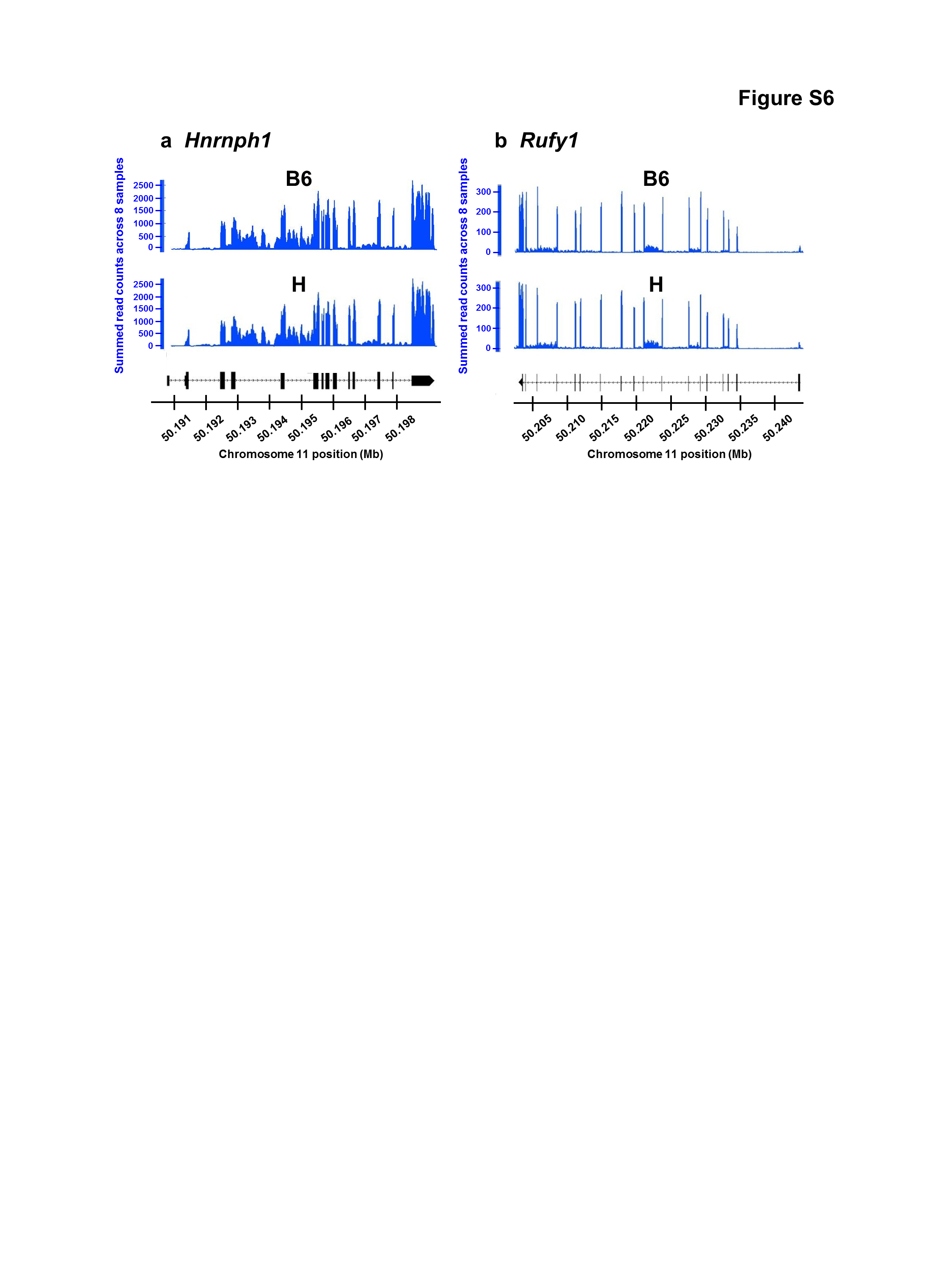

Supplement: S6 Fig — (a, b): The x-axis represents the physical location (bp) of the annotated exons (vertical lines, UCSC Genome Browser; mm9) on chromosome 11 for Hnrnph1 and Rufy1. The y-axis represents the summed read counts (y-axis) across all 8 samples for each genotype (B6, H). Note that different scales are used on the y-axis for Hnrnph1 (the more highly expressed gene; 0–2500 reads) versus Rufy1 (0–300 reads). (TIF) [file pgen.1005713.s017.tif]

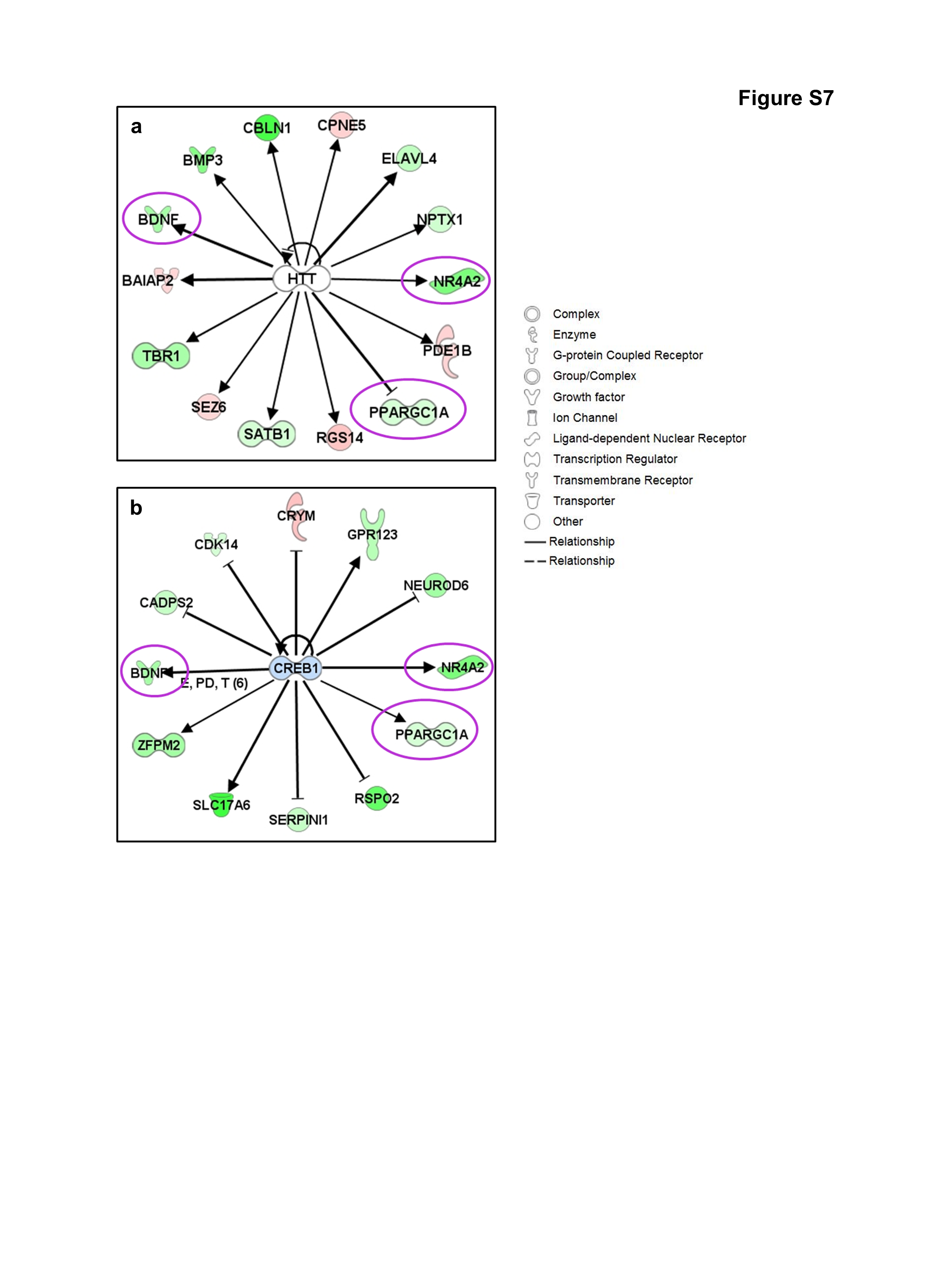

Supplement: S7 Fig — Arrows pointing toward genes indicate predicted activation; horizontal, perpendicular lines indicate predicted inhibition. Green and red colors indicate downregulated or upregulated genes in our dataset. Purple circles denote genes that overlap between Htt (a) and Creb1 (b). The legend on the right hand side denotes the biological classification for each gene contained in the regulator diagrams. (TIF) [file pgen.1005713.s018.tif]

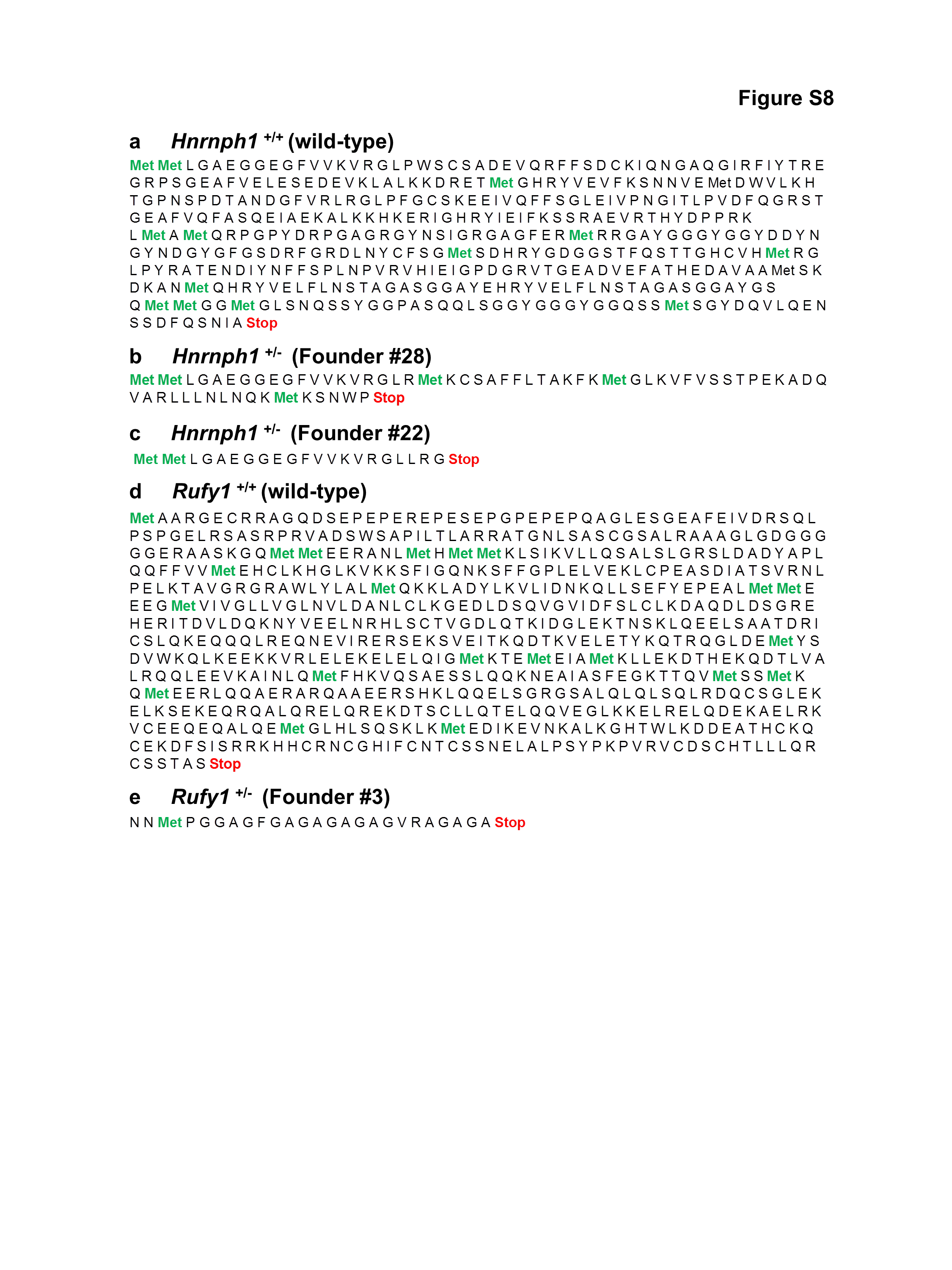

Supplement: S8 Fig — We used the ExPASy Translate Tool (http://web.expasy.org/translate/) to input wild-type and deleted cDNA sequences to obtain protein sequences. (a-c): Amino acid sequence is shown for Hnrnph1 +/+ mice and Hnrnph1 +/- founders. (d, e): Amino acid sequence is shown for Rufy1 +/+ and Rufy1 +/- founders. Methionine (Met) is shown in green. A red “Stop” denotes a stop codon. (TIF) [file pgen.1005713.s019.tif]

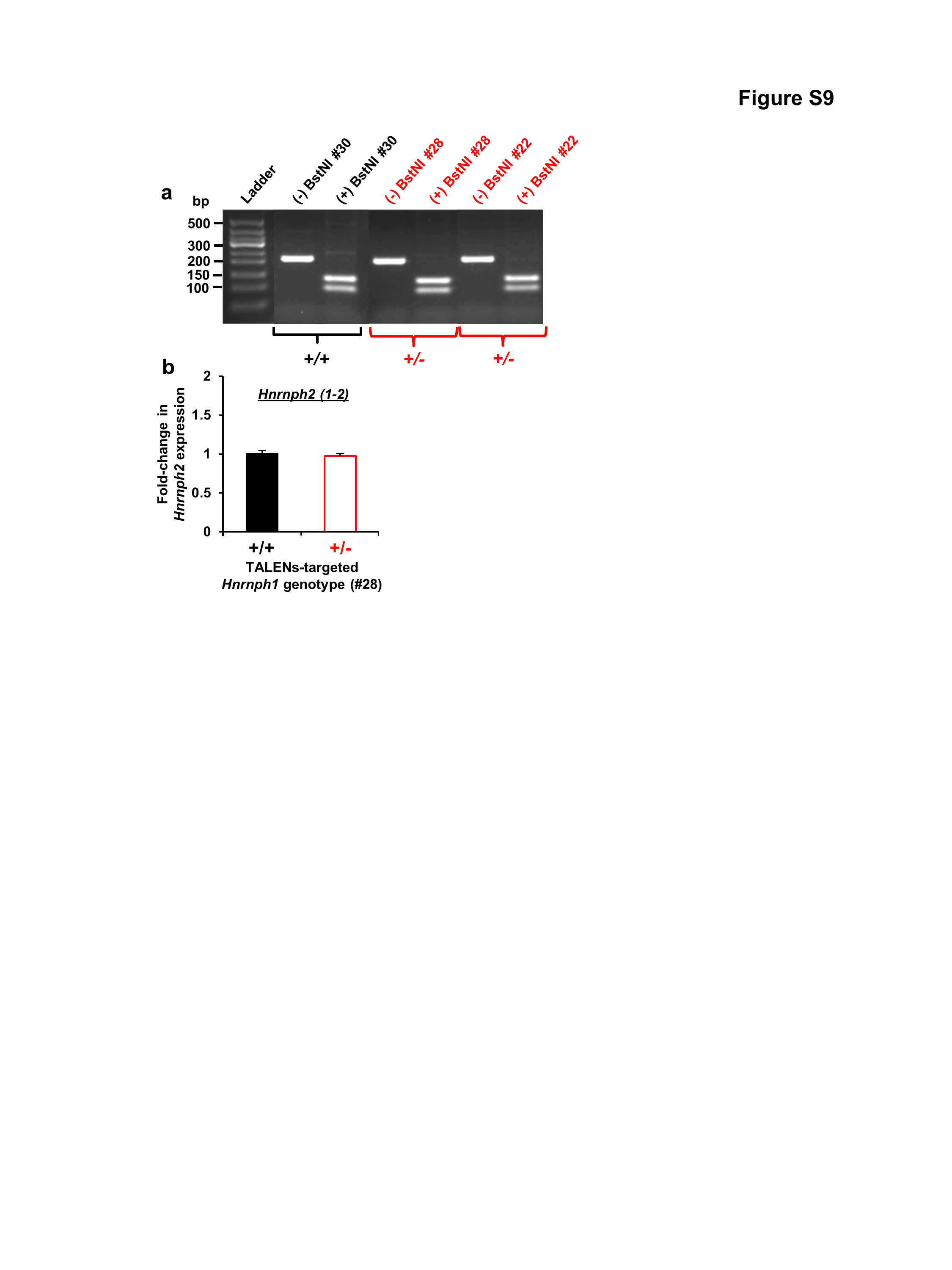

Supplement: S9 Fig — (a): A 197 bp PCR amplicon was generated using primers specific for exon 4 of Hnrnph2 and contained the same homologous BstNI cut site as exon 4 in Hnrnph1 (Fig 5). Hnrnph1 +/+ mice and Hnrnph1 +/- founder mice (#28 and #22) that were heterozygous for an Hnrnph1 frameshift deletion all showed two bands following restriction digest, indicating that there was no deletion of the restriction site in Hnrnph2. (b): There was no compensatory change in Hnrnph2 expression in Line #28 when comparing Hnrnph1 +/- (N = 4) versus Hnrnph1 +/+ (N = 4) mice (t6 < 1). Data are presented as the mean ± S.E.M. (TIF) [file pgen.1005713.s020.tif]

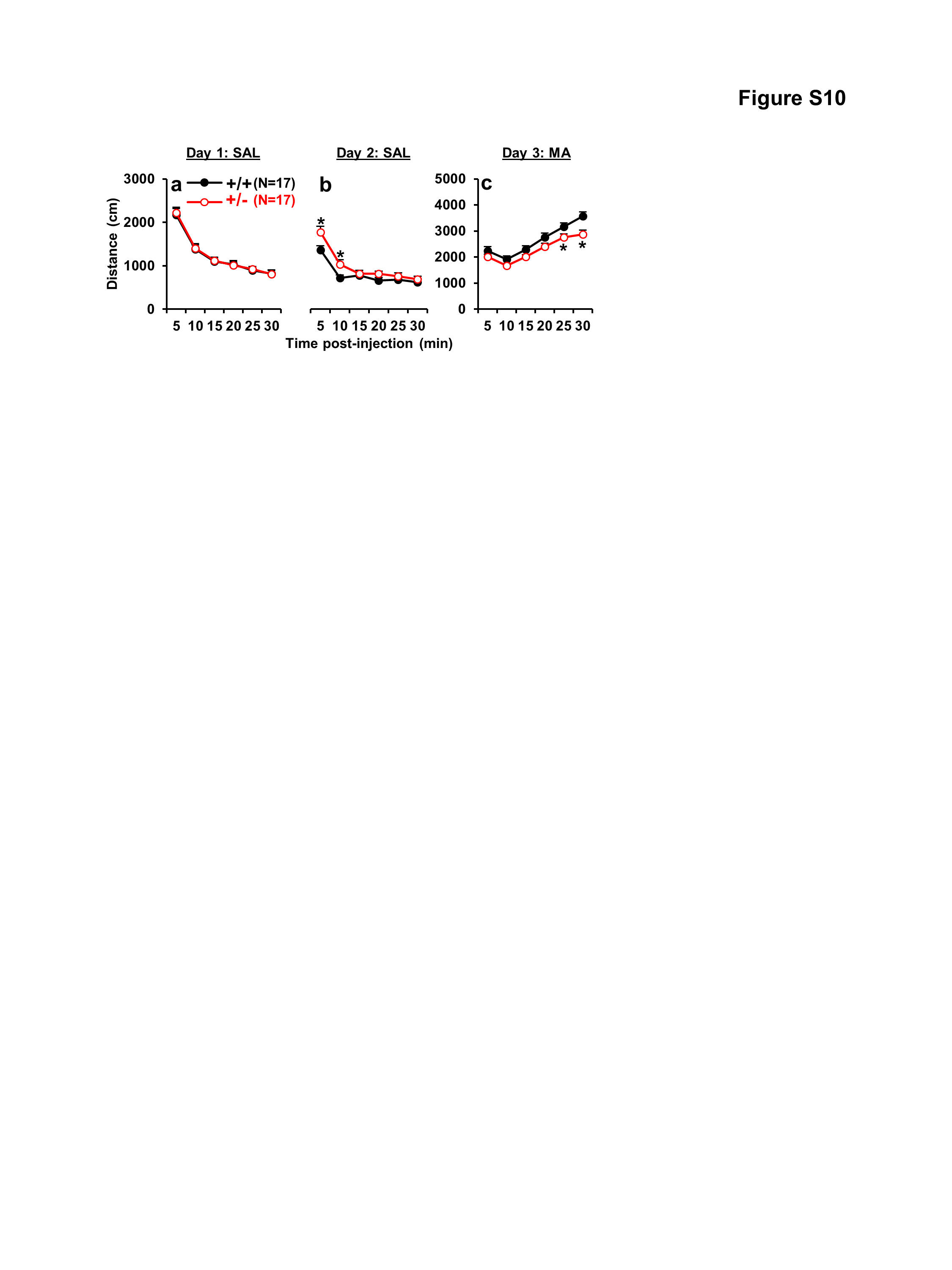

Supplement: S10 Fig — (a): For Day 1, there was no effect of genotype (F1,32 < 1) nor any interaction with time (F5,160 <1). (b): For Day 2, there was no effect of genotype (F1,32 = 3.79; p = 0.06) but there was a significant genotype x time interaction (F5,160 = 3.66; p = 0.0037 that was explained by Hnrnph 1 +/- mice showing significantly greater locomotor activity than Hnrnph1 +/+ mice at the 5-min and 10-min time bins (t32 = 2.53, 2.42; p = 0.017, 0.021). (c): For Day 3, there was an effect of genotype (F1,32 = 5.37; p = 0.027) but no significant genotype x time interaction (F5,160 = 2.04; p = 0.076). Hnrnph1 +/- mice showed significantly less MA-induced locomotor activity than Hnrnph1 +/+ mice at 25 and 30 min (t32 = 2.07, 3.03; p = 0.046, 0.0048). Data are presented as the mean ± S.E.M. *p < 0.05. (TIF) [file pgen.1005713.s021.tif]

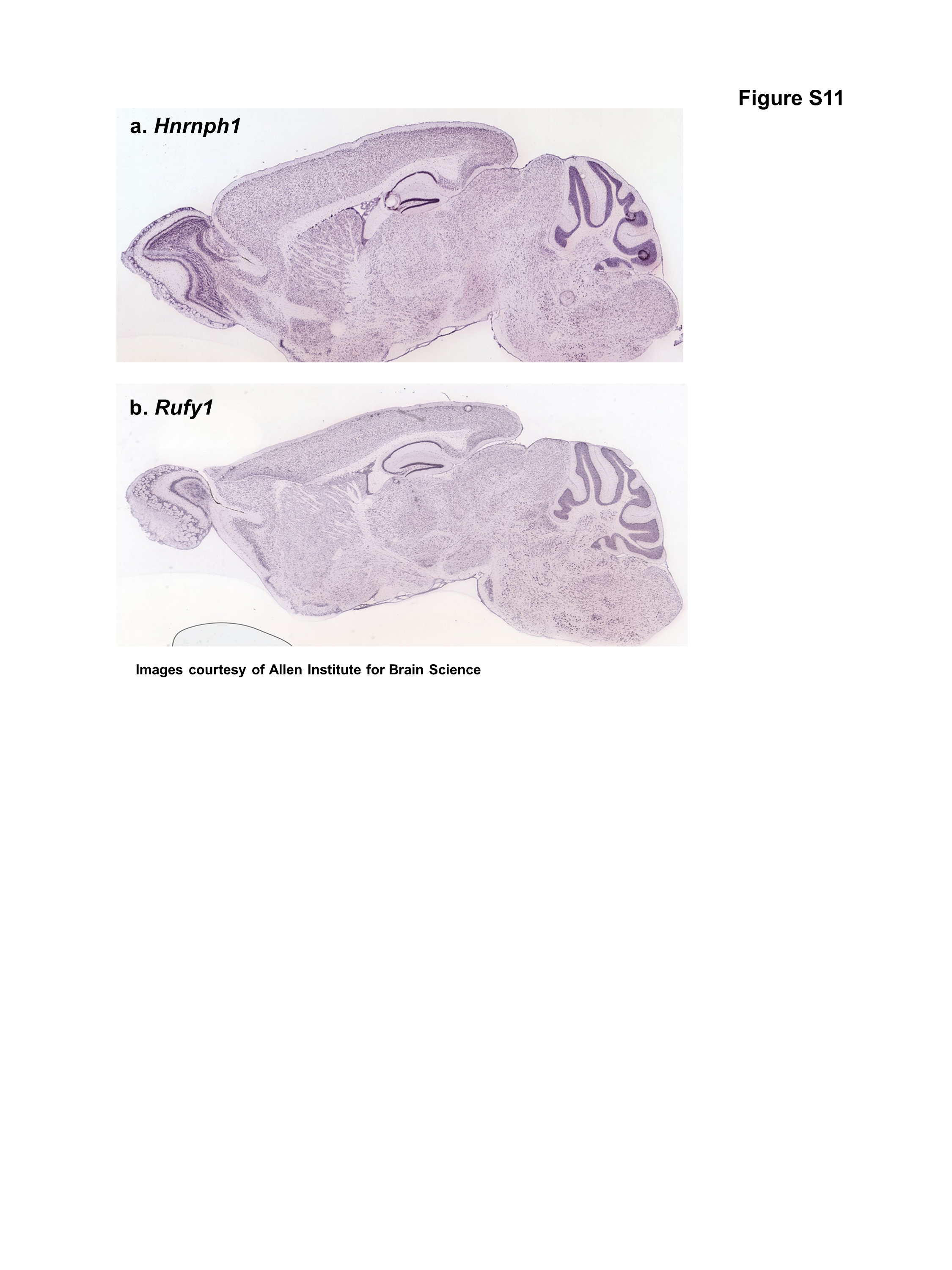

Supplement: S11 Fig — In situ hybridization staining of mid-sagittal sections are shown for Hnrnph1 (panel a) and Rufy1 (panel b) and were obtained from the Allen Institute for Brain Science (http://www.brain-map.org/ 4). Hnrnph1 clearly shows higher expression than Rufy1 which can also evident in the number of read counts in our dataset (see also S6 Fig). (TIF) [file pgen.1005713.s022.tif]
